# Fis Is Essential for *Yersinia pseudotuberculosis* Virulence and Protects against Reactive Oxygen Species Produced by Phagocytic Cells during Infection

**DOI:** 10.1371/journal.ppat.1005898

**Published:** 2016-09-30

**Authors:** Erin R. Green, Stacie Clark, Gregory T. Crimmins, Matthias Mack, Carol A. Kumamoto, Joan Mecsas

**Affiliations:** 1 Graduate Program in Molecular Microbiology, Sackler School of Graduate Biomedical Sciences, Tufts University School of Medicine, Boston, Massachusetts, United States of America; 2 Department of Molecular Biology and Microbiology, Tufts University School of Medicine, Boston, Massachusetts, United States of America; 3 Universitatsklinikum Regensburg, Innere Medizin II/Nephrologie-Transplantation, Regensburg, Germany; University of California Davis School of Medicine, UNITED STATES

## Abstract

All three pathogenic *Yersinia* species share a conserved virulence plasmid that encodes a Type 3 Secretion System (T3SS) and its associated effector proteins. During mammalian infection, these effectors are injected into innate immune cells, where they block many bactericidal functions, including the production of reactive oxygen species (ROS). However, *Y*. *pseudotuberculosis* (*Yptb*) lacking the T3SS retains the ability to colonize host organs, demonstrating that chromosome-encoded factors are sufficient for growth within mammalian tissue sites. Previously we uncovered more than 30 chromosomal factors that contribute to growth of T3SS-deficient *Yptb* in livers. Here, a deep sequencing-based approach was used to validate and characterize the phenotype of 18 of these genes during infection by both WT and plasmid-deficient *Yptb*. Additionally, the fitness of these mutants was evaluated in immunocompromised mice to determine whether any genes contributed to defense against phagocytic cell restriction. Mutants containing deletions of the *dusB-fis* operon, which encodes the nucleoid associated protein Fis, were markedly attenuated in immunocompetent mice, but were restored for growth in mice lacking neutrophils and inflammatory monocytes, two of the major cell types responsible for restricting *Yersinia* infection. We determined that Fis was dispensable for secretion of T3SS effectors, but was essential for resisting ROS and regulated the transcription of several ROS-responsive genes. Strikingly, this protection was critical for virulence, as growth of *ΔdusB-fis* was restored in mice unable to produce ROS. These data support a model in which ROS generated by neutrophils and inflammatory monocytes that have not been translocated with T3SS effectors enter bacterial cells during infection, where their bactericidal effects are resisted in a Fis-dependent manner. This is the first report of the requirement for Fis during *Yersinia* infection and also highlights a novel mechanism by which *Yptb* defends against ROS in mammalian tissues.

## Author Summary

The pathogenic members of the genus *Yersinia* share a conserved virulence plasmid that primarily serves to encode a Type 3 Secretion System and its associated effector proteins. During mammalian infection, these effectors are targeted toward phagocytic cells, where they neutralize a multitude of functions, including oxidative burst. However, it has previously been reported that strains of *Yersinia pseudotuberculosis* lacking the virulence plasmid retain the ability to grow in mammalian tissue sites, suggesting that the *Yersinia* chromosome encodes a number of poorly appreciated factors that enable survival in mammalian tissue sites, even in the absence of a functional T3SS. Here, we further characterize a number of these factors, including the operon *dusB-fis*. Using a variety of *in vitro* and *vivo* approaches, we determined that Fis regulates the transcription of several genes implicated in ROS resistance and that *dusB-fis* is essential for preventing growth restriction by ROS produced by the NADPH complex of phagocytes, even in a T3SS-expressing strain. Combined, these data suggest a model in which, during tissue infection, *Yersinia* evade killing by ROS through both T3SS-dependent and independent mechanisms.

## Introduction

Bacterial pathogens utilize both “defensive” and “offensive” strategies to survive in mammalian tissue sites and withstand the host immune response [1]. “Defensive” strategies often consist of physiological adaptations to stresses encountered in tissues, such as changes in pH or temperature, nutrient restriction, or influxes of toxic gases or proteins released by immune cells [2,3]. Many of these stresses are also found in other environments pathogens inhabit, such as soil, fomites, or in aerosol particles. By contrast, “offensive” strategies include the secretion of toxins or effector proteins that kill or block the actions of responding immune cells [1]. One such example is the Type 3 Secretion System (T3SS) used by many bacterial pathogens, including *Yersinia*, *Salmonella*, *Shigella*, *Pseudomonas*, and *Chlamydia* [4]. These systems translocate effector proteins into mammalian cells from the bacterial cytosol and, often, promote bacterial growth by neutralizing the anti-bacterial actions of these cells. Additionally, T3SS effectors are used by some pathogens to rearrange host cell processes to enable intracellular growth [4]. In other cases, effector proteins act to kill mammalian cells by targeting essential proteins or triggering cell death pathways [4].

T3SS effector proteins play critical roles in the virulence of the pathogenic *Yersinia* species, which include the pneumonic and bubonic agent *Yersinia pestis*, as well as the gastrointestinal pathogens *Yersinia enterocolitica* and *Yersinia pseudotuberculosis (Yptb)* [5]. These three organisms target the translocation of their T3SS effectors, called Yops, into responding phagocytic cells, particularly into neutrophils, where they dismantle a number of bactericidal responses, including the ability to phagocytose bacteria, release reactive oxygen species (ROS), and produce certain inflammatory cytokines [5–11]. The contributions of the T3SS and Yops to *Yersinia* pathogenesis have been extensively studied for more than two decades, and a number of reports have been published on Yop targets and their actions in mammalian cells [5,12]. However, during infection of mammalian tissue sites, not all immune cells are intoxicated with Yops [13,14], indicating that *Yersinia* must also employ additional, T3SS-independent strategies for surviving within the host and resisting the immune response, as remaining, non-intoxicated immune cells are competent to execute bactericidal functions. For example, it is known that at least one of these bactericidal functions, release of nitric oxide, restricts bacterial growth from a distance by cells not directly intoxicated with Yops [15]. Furthermore, *Yptb* lacking the pIB1 virulence plasmid, which encodes the T3SS and Yops, is capable of infecting and replicating within mouse tissues, in some cases at levels equivalent to a WT strain [16–18]. Even though infection with this strain seldom leads to death, these findings indicate that T3SS-deficient *Yptb* can withstand host defenses for several days and thus, likely encode “defensive” factors on its chromosome that allow for survival in harsh environments.

Indeed, some of these genes have been identified in high throughput screens in various *Yersinia* species [19–24], but all of these screens were performed in the presence of the virulence plasmid, which may mask the functions of some chromosomal factors. To determine which chromosomal factors contribute to infection of *Yptb* in the absence of the T3SS, we previously screened a library of 20,000 transposon insertions in a plasmid-deficient (pIB1^-^) *Yptb* strain during systemic infection and identified more than 30 mutants that were attenuated in livers [17]. One of these mutants, Δ*mrtAB*, was attenuated for virulence in the absence of the T3SS in all tissues, but only attenuated in the mesenteric lymph nodes in the presence of the T3SS, indicating that this screen uncovered factors that are redundant with the T3SS and/or that pIB1^+^ and pIB1^-^ *Yptb* encounter distinct environments in some tissue sites [17].

Here, we follow up on our original work by employing a high-throughput, sequencing-based assay to evaluate the contribution of 18 additional genes identified in the screen to infection by both a plasmid-deficient and WT *Yptb*. In contrast to our findings with Δ*mrtAB*, we found that most of the genes evaluated were important for systemic infection by both pIB1^+^ and pIB1^-^ *Yptb*, indicating that these genes play essential roles in virulence, regardless of the T3SS. Additional testing of the mutants in immunocompromised mice demonstrated that 4 loci were critical for virulence when interfacing with phagocytic cells. One operon, *dusB-fis*, prevented growth restriction by phagocytes and killing by ROS both *in vitro* and *in vivo*, in the presence of the T3SS. This work highlights the importance of studying both “offensive” (T3SS-dependent) and “defensive” (T3SS-independent) mechanisms of survival during *Yersinia* infection models, as both strategies are essential for the establishment of virulent infection.

## Results

### Mutants evaluated in mini-TnSeq assay were significantly defective for virulence in both T3SS^-^ and T3SS^+^ strains of *Yptb*

By evaluating the virulence of 20,000 transposon mutants in a plasmid-deficient *Yptb* strain, we identified 33 mutants that were significantly defective for colonization of and/or growth within the liver, but were otherwise capable of growing in rich media at physiological temperatures [17]. However, using large pools of transposon mutants in animal infection models can sometimes result in “false negatives,” as libraries are subject to bottleneck constraints and transposon insertions can have polar effects on nearby loci [25]. Therefore, we devised and implemented a high-throughput, sequencing-based approach (a “mini” TnSeq) to simultaneously compare the survival of multiple in-frame deletion mutants in small infection pools to further evaluate the loci identified in our previous TnSeq screen (S1 Fig). Eighteen gene or operon deletion mutants containing identical in-frame scar sequences (S1A Fig) were generated in both a plasmid-deficient (pIB1^-^) YPIII strain (the parental strain of our original transposon library) and a plasmid-containing (pIB1^+^) IP2666 strain to determine whether the *in vivo* contributions of some of these genes may be influenced by the T3SS. We chose the IP2666 pIB1^+^ strain for further investigation because it encodes the known virulence factor, *phoP*, which is non-functional in YPIII due to a mutation [26]. The 18 operons and genes examined represent several broad functional classes, including biosynthesis of metabolic compounds, LPS synthesis and modification, and several other previously uncharacterized virulence factors (Table 1). In order to ensure that the bacterial pools used to infect mice contained an equal proportion of attenuated mutants and WT bacteria, we also constructed two deletions of “neutral genes” (Table 1), which were selected because transposon disruptions in these genes had no deleterious effects in the original TnSeq screen [17].

**Table 1:** Mutants in mini-TnSeq assay.

	pIB1^-^		pIB1^+^	
Strains^1^	Liver	Spleen	Liver	Spleen
**Metabolism**				
*ΔaroA*	**** ^2^	ns	****	ns
*ΔaroE*	****	****	****	****
*ΔpurM*	****	****	****	****
**LPS**				
*ΔYPK_3179*	****	****	***	***
*ΔYPK_3184*	****	**	**	ns
*ΔYPK_3185*	ns	ns	ns	ns
*ΔrfaH*	****	**	*	**
*ΔwecC*	****	ns	**	****
*ΔarnDT*	***	ns	****	ns
**Other**				
*ΔdusB-fis*	****	***	**	****
*ΔYPK_1920*	****	ns	****	ns
*ΔYPK_2066*	*	ns	ns	ns
*ΔflgD*	ns	ns	ns	ns
*ΔYPK_2594*	**	ns	****	ns
*ΔpsaEFABC*	****	****	*	ns
*ΔYPK_3600*	ns	ns	ns	ns
*ΔYPK_3656*	ns	ns	ns	ns
*ΔYPK_3765*	****	****	**	****
**Neutral**				
*ΔYPK_1604*	N/A	N/A	N/A	N/A
*ΔYPK_2061*	N/A	N/A	N/A	N/A

^1^ genes or operons deleted in the mini-TnSeq screen

^2^ p-values representing comparisons between the fitness scores of individual mutants evaluated in mini-TnSeq assay and the fitness score of the two neutral mutants (pooled together), as described in Fig 1 and Experimental Procedures.

* indicates p≤0.05,

** indicates p≤0.01,

*** indicates p≤0.001,

**** indicates p≤0.0001, ns indicates not significant p>0.05.

N/A indicates not applicable.

**Fig 1:**
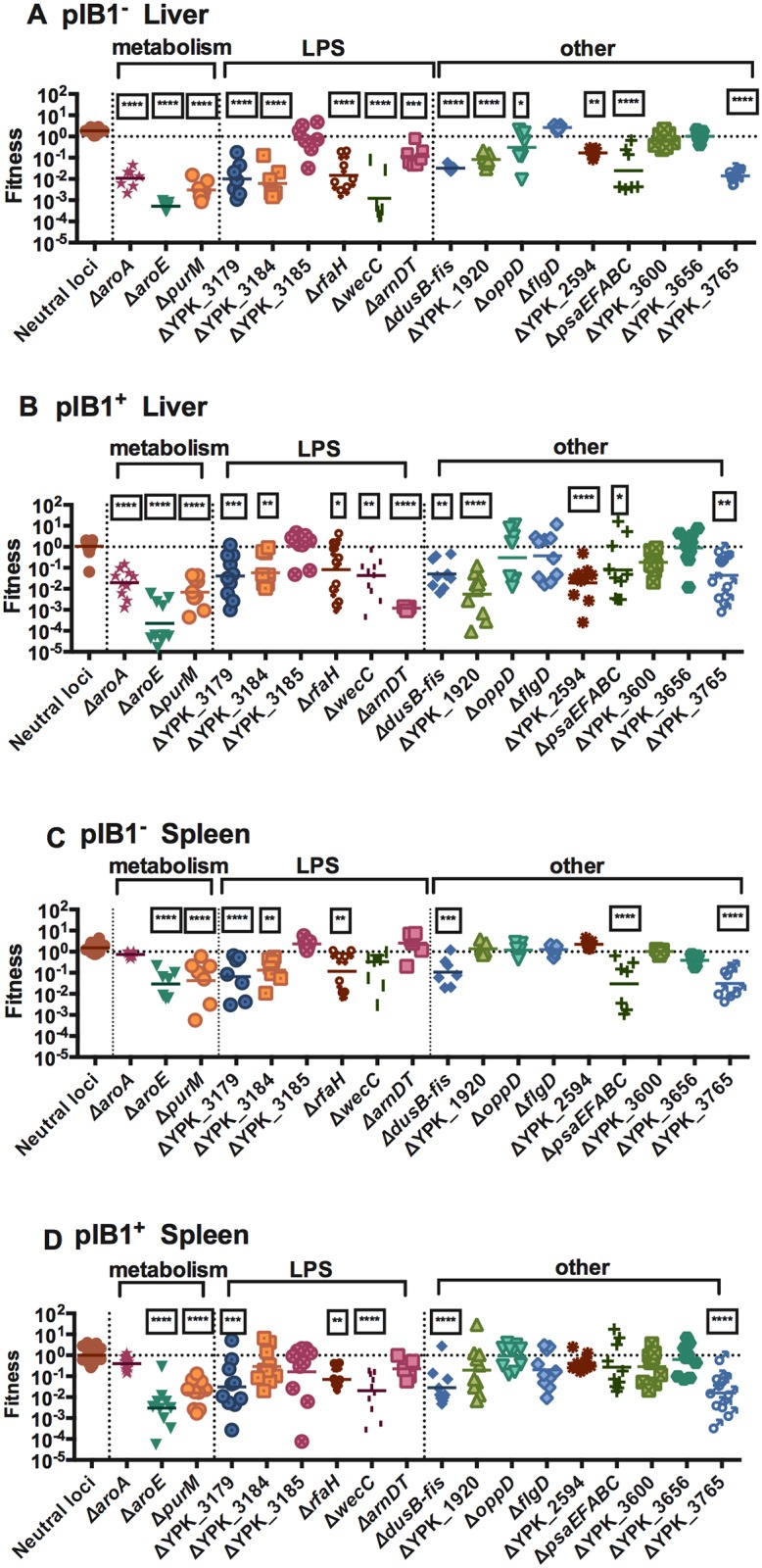
Virulence of mutants in mini-TnSeq assay. Fitness of knockouts generated in YPIII/pIB1^-^ (A, C) or IP2666/pIB1^+^ (B, D) at 3 days post-intravenous infection with 10^4^ and 10^3^, respectively, of mini-TnSeq libraries. Fitness values were obtained by dividing the proportion of sequencing reads for a mutant in the liver (A, B) or spleen (C, D) by its proportion of reads in the inoculum. Each data point for a mutant represents an individual mouse. N = 7–10 mice. Fitness values were log_10_ transformed and statistical significance was calculated using One Way ANOVA analysis with Dunnett’s multiple comparison post-test. P-values represent comparisons between the fitness scores of individual mutants with the fitness score of the two knockouts of neutral loci (pooled together) in each respective condition. * indicates p≤0.05, ** indicates p≤0.01, *** indicates p≤0.001, and **** indicates p≤0.0001.

Mice were infected intravenously with 10^4^ or 10^3^ CFU of the pIB1^-^ or the pIB1^+^ library, respectively. Following infection with these doses, equivalent bacterial loads were recovered from spleens at 3 days post-infection regardless of the presence of the T3SS (S2A Fig); however, livers infected with pIB1^-^ bacteria contained lower bacterial loads than those infected with libraries generated in the WT background (S2B Fig). In each pool, the two neutral strains each comprised 25% of the inoculum, while the remaining 18 mutants each comprised ~3% of the population. Following recovery of bacteria from infected livers and spleens at 3 days post-infection, genomic DNA was processed for Illumina sequencing and fitness values were calculated for each mutant (Fig 1). Strikingly, 14 of the 18 mutants generated in the pIB1^-^ YPIII background had statistically significant virulence defects in infected liver tissues. Of those 14 genes, all but one were also critical for growth of pIB1^+^ IP2666 within the liver (Fig 1A and 1B, Table 1), indicating that more than 70% of the genes evaluated were important for infection, regardless of the presence of the T3SS. Mutants attenuated for growth within the liver included the auxotrophic strains Δ*aroA* and Δ*aroE*, which are unable to produce aromatic amino acids [27,28], and Δ*purM*, which lacks a component of the purine biosynthesis pathway [29]. With the exception of one strain (Δ*YPK_3185*), all of the strains with mutations in genes involved with LPS synthesis and modification were attenuated for virulence in at least one tissue site. Importantly, several factors that had not been previously characterized in *Yersinia* infection models, including *YPK_2594*, which has no predicted function, *YPK_1920*, which is predicted to encode a lipoprotein, *YPK_3765*, which is predicted to encode a murein peptide ligase, and the *dusB-fis* operon, which encodes the nucleoid associated protein Fis, were critical for infection. Six pIB1^+^ mutants, Δ*aroA*, Δ*YPK_3184*, Δ*arnDT*, Δ*YPK_1920*, Δ*YPK_2594*, and Δ*psaEFABC*, were defective for growth in the liver (Fig 1B), but not the spleen (Fig 1D and Table 1), indicating tissue specific functions of these genes.

**Fig 2:**
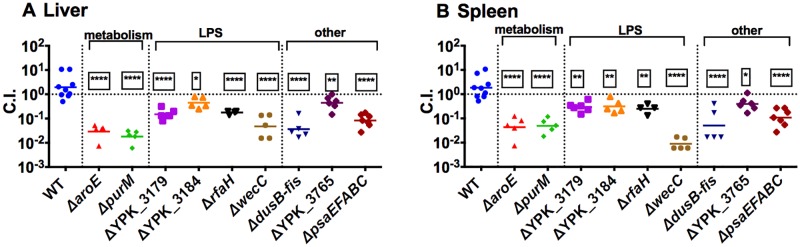
Mini-TnSeq accurately predicted outcomes of 1:1 competition experiments. Mice were inoculated intravenously with 1:1 mixture of 10^3^ bacteria, comprised of WT-Kan^R^ and a drug-sensitive mutant. Livers (A) and spleens (B) were dissected at 3 days post-infection, homogenized and plated onto selective and non- selective plates to determine the competitive index (C.I.). Each symbol represents an individual mouse. N = 4–10. C.I. data was log_10_ transformed and statistical significance was calculated using One Way ANOVA analysis with Dunnett’s multiple comparison post-test comparing the C.I. values of mutant strains with those of the WT strain. * indicates p≤0.05, ** indicates p≤0.01, *** indicates p≤0.001, and **** indicates p≤0.0001.

To evaluate whether mutants were attenuated when they were not a small minority of the input pool, traditional competition experiments were performed using bacterial mutants generated in the pIB1^+^ background. Mutants were mixed at a 1:1 ratio with a drug resistant WT strain, and C.I. values were obtained after intravenous infection (Fig 2). All of the mutants evaluated were attenuated in this assay. In conclusion, using our efficient and highly sensitive mini-TnSeq assay many bacterial mutants were attenuated in both WT and plasmid-deficient *Yptb*, indicating that most of these loci do not have redundant roles with the T3SS.

### Growth of Δ*dusB-fis*, Δ*YPK_3765*, Δ*rfaH*, and Δ*psaEFABC* mutants is altered in immunocompromised mice

**Fig 3:**
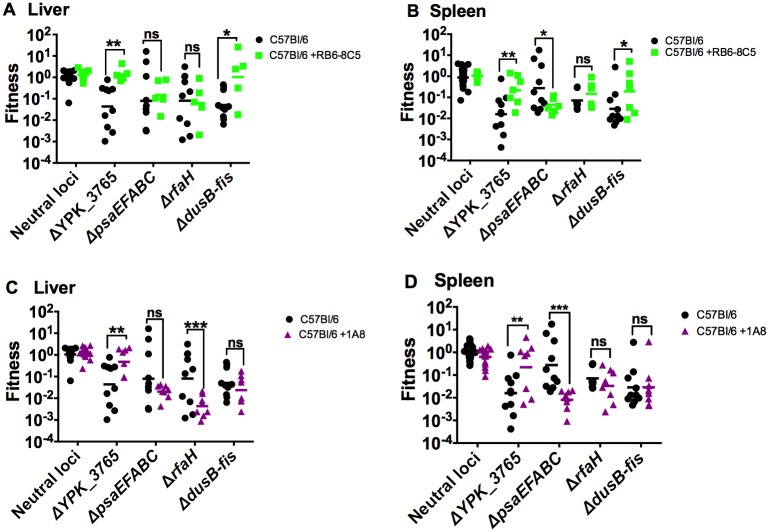
Four mutants displayed fitness changes following phagocytic cell depletion. Mice were intraperitoneally injected with RB6-8C5 (A-B) or 1A8 (C-D) 24 hours prior to and post-infection. Mice were inoculated intravenously with 10^3^ CFU of the IP2666/pIB1^+^ mutant library. Fitness values were obtained by dividing the proportion of sequencing reads for a mutant in the depleted liver (A, C) or spleen (B, D) by its proportion of reads in the inoculum. Each data point for a mutant represents an individual mouse. N = 5–10 mice. Non-depleted fitness values are the same is in Fig 1. Fitness scores values were log_10_ transformed and an unpaired t-test with the Holm-Sidak correction for multiple comparisons was performed to calculate statistically significant differences between the fitness scores of specific bacterial mutants in depleted versus non-depleted mice. * indicates p≤0.05, ** indicates p≤0.01, *** indicates p≤0.001, and **** indicates p≤0.0001.

Infection with *Yptb* produces a pronounced inflammatory response, where bacteria growing in tissue sites are surrounded by phagocytic cells [15–17]. Therefore, we hypothesized that some of the genes evaluated in the mini-TnSeq assay may encode proteins that directly interface with phagocytic cells, or are important for surviving in the face of anti-microbial responses generated by these cells. To test this, mice pre-treated with the RB6-8C5 antibody, which depletes Gr1^pos^ cells (Ly6G^pos^ neutrophils and Ly6C^pos^ inflammatory monocytes, dendritic cells, and lymphocytes) or the 1A8 antibody, which depletes Ly6G^pos^ cells (neutrophils only) were infected with the pIB1^+^ mini-TnSeq library. Surprisingly, very few significant changes in fitness scores were detected following infection of immunocompromised mice with these mutants (S3 Fig), suggesting that most of these genes are important for bacterial colonization and growth in animal tissues, regardless of the presence of these innate immune cells. However, four mutants displayed significantly altered fitness scores upon infection of immunocompromised mice (Fig 3). Growth of Δ*YPK_3765* was restored to WT levels in the livers and spleens of both RB6-8C5- and 1A8-treated mice (Fig 3A–3D), indicating that this gene is required for resisting growth restriction by phagocytic cells. Surprisingly, depletion with the RB6-8C5 and 1A8 antibodies resulted in decreased growth of the Δ*psaEFABC* mutant in spleens (Fig 3B and 3D), and depletion with 1A8 decreased the growth of Δ*rfaH* in livers (Fig 3A and 3C). These results suggest that neutrophils may protect these mutants from further growth restriction by other cells or factors in these tissue sites. Interestingly, the growth changes observed with Δ*YPK_3765*, Δ*rfaH*, and Δ*psaEFABC* were specific to neutrophil depletion, as treatment with 1A8 was sufficient to alter the fitness of these mutants. By contrast, the fitness of Δ*dusB-fis* was restored in mice treated with the RB6-8C5 antibody (Fig 3A and 3B), but not in mice treated with the 1A8 antibody (Fig 3C and 3D). This result suggested that Δ*dusB-fis* is sensitive to all Gr1^pos^ cells, to one or more Ly6C^pos^ cell types (inflammatory monocytes, dendritic cells, and lymphocyte subsets), or to Ly6G^pos^ neutrophils and a subset of Ly6C^pos^ cells.

**Fig 4:**
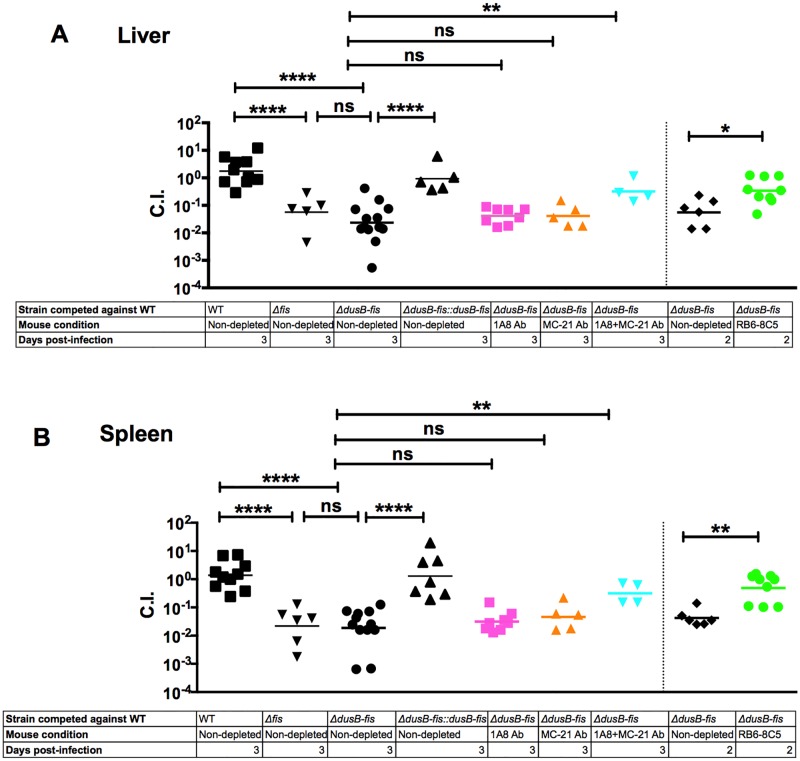
*ΔdusB-fis* is sensitive to neutrophils and inflammatory monocytes *in vivo*. C57BL6/J mice were inoculated intravenously with a pool of 10^3^ bacteria, containing an equal mixture of WT and WT*-*Kan^R^, *Δfis-*Kan^R^ and WT, *ΔdusB-fis* and WT*-*Kan^R^, or *ΔdusB-fis*::*dusB-fis* and WT*-*Kan^R^. 24 hours prior to and post-infection, some mice were intraperitoneally injected with either the 1A8 or RB6-8C5 antibody. Other mice were intraperitoneally injected with the MC-21 antibody 1 day prior to infection and each day after until completion of the experiment. Mice were euthanized at the indicated times and livers (A) and spleens (B) were collected, and dilutions of tissue homogenates were plated onto selective and non-selective media to determine the C.I. C.I. data was log_10_ transformed and statistical significance was calculated using One Way ANOVA analysis with Dunnett’s multiple comparison post-test comparing the C.I. values of mutant strains to WT or C.I. values in non-depleted and depleted mice. * indicates p≤0.05, ** indicates p≤0.01, *** indicates p≤0.001, and **** indicates p≤0.0001.

To distinguish among these possibilities, we performed 1:1 co-infection experiments with Δ*dusB-fis* in mice treated with an antibody, MC-21, which blocks the chemokine receptor CCR2 and prevents recruitment of Ly6C^pos^ inflammatory monocytes to tissue sites during microbial infection [30–33]. Additional cohorts of mice were treated with 1A8, RB6-8C5, or with a combination of the 1A8 and MC-21 antibodies prior to infection. Depletion of cell subsets was confirmed by flow cytometry using Gr1 and Cd11b markers to distinguish between neutrophil and inflammatory monocyte populations (S4 Fig). Treatment with either 1A8 or MC-21 alone did not restore the virulence of the Δ*dusB-fis* mutant (Fig 4), indicating that the presence of either Ly6G^pos^ or Ly6C^pos^ cell type(s) at the site of infection was sufficient to restrict the growth of this mutant. By contrast, depletion with a combination of the MC-21 and 1A8 antibodies restored growth of Δ*dusB-fis* in livers and spleens, demonstrating that Δ*dusB-fis* is specifically sensitive to neutrophils and CCR2-recruited inflammatory monocytes during tissue infection. Importantly, growth of Δ*dusB-fis* was also restored when we complemented this mutant by re-introducing the *dusB*-*fis* genes into the deleted strain (Fig 4). To determine whether a *dusB-fis* mutant was more attenuated than *fis*, a deletion of *fis* was generated and evaluated in mice. The *fis* mutant was attenuated to the same extent as Δ*dusB-fis* (Fig 4A and 4B), indicating that Fis is essential for the virulence of *Yptb*. In summary, these results demonstrate that Fis promotes *Yptb* resistance to or evasion of killing by both neutrophils and inflammatory monocytes during mouse infection.

### The *dusB-fis* mutant colonizes systemic sites efficiently, but is restricted for growth after colonization

**Fig 5:**
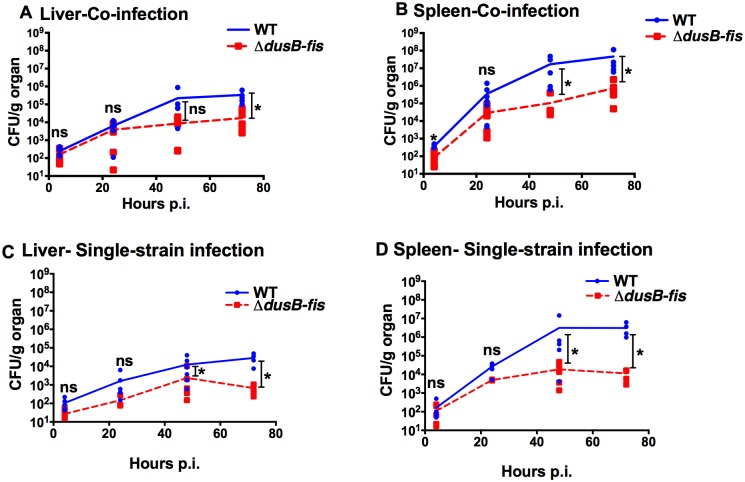
*ΔdusB-fis* can colonize, but is unable to sustain growth in systemic tissue sites. C57BL6/J mice were inoculated intravenously with a pool of 10^3^ bacteria, containing either an equal mixture of WT *yopE*::*mcherry* and *ΔdusB-fis-*Kan^R^ (A-B), or with WT *yopE*::*mcherry* only or *ΔdusB-fis-*Kan^R^ only (C-D). At 4-hour, 24-hour, 48-hour, and 72-hour time-points post-infection, mice were euthanized and spleens and livers were collected. Dilutions of liver (A, C) and spleen (B, D) homogenates were plated and CFU calculated. In co-infection experiments (A-B), the number of bacteria recovered from selective and non-selective plates was used to determine quantity of WT *yopE*::*mcherry* and *ΔdusB-fis-*Kan^R^ bacteria in each organ sample. CFU/g data was log_10_ transformed and the values of *ΔdusB-fis-*Kan^R^ to WT *yopE*::*mcherry* bacteria in each tissue at each time point was compared using an unpaired t-test with the Holm-Sidak correction for multiple comparisons. * indicates p≤0.05, ** indicates p≤0.01, *** indicates p≤0.001, and **** indicates p≤0.0001.

Because the virulence of the Δ*dusB-fis* mutant was restored in the absence of neutrophils and inflammatory monocytes, it is possible that these immune cells restricted survival of this mutant in the bloodstream after intravenous infection, thereby preventing high levels of tissue colonization. Alternatively, or in addition, neutrophils and inflammatory monocytes may restrict the growth of Δ*dusB-fis* in the systemic tissue sites once these cells surround the bacteria. To distinguish between these two possibilities, the growth kinetics of the Δ*dusB-fis* mutant were determined during systemic mouse infection at 4, 24, 48, and 72-hour time-points. In both co-infections with a WT strain (Fig 5A and 5B) and in single-strain infections (Fig 5C and 5D), the Δ*dusB-fis* mutant colonized tissue sites and grew for the first 24 hours post-infection with kinetics similar to WT *Yptb*. However, by 48 hours post-infection, the level of Δ*dusB-fis* failed to increase as rapidly as WT, indicating that the growth of this strain was not restricted until after initial seeding and expansion in tissue sites. Combined with our findings from the depletion experiments, these results suggest that Δ*dusB-fis* cannot adapt to a change in the tissue environment that likely occurs due to the influx and/or activities of neutrophils and inflammatory monocytes.

### *The dusB-fis* operon is critical for resisting oxidative stresses and regulates the transcription of ROS-responsive genes

**Fig 6:**
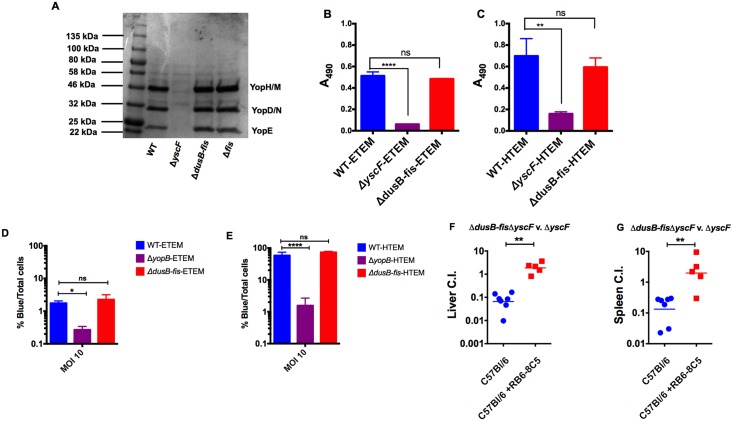
*ΔdusB-fis* is not defective for Type 3 Secretion or effector translocation. A) Stationary phase cultures of WT, *ΔyscF*, Δ*dusB-fis*, and Δ*fis* strains were diluted 1:40 into L broth with 20mM sodium oxalate and 20 mM MgCl_2_. Cultures were grown for 2 hours at 26°C and then shifted to 37°C for 2 hours. After growth, proteins from culture supernatants were precipitated, resolved on a polyacrylamide gel alongside a standard protein ladder, and visualized using commaasie blue. B-C) Stationary phase cultures of WT, *ΔyscF*, and Δ*dusB-fis* strains containing YopH-TEM (HTEM) or YopE-TEM (ETEM) beta-lactamase reporters were grown as in A. After growth, the culture supernatant was mixed with nitrocefin at a final concentration of 100 μg/mL. After 10 minutes of incubation, the A_490_ of samples was measured using a BioTek Synergy HT plate reader. Statistical significance was calculated using One Way ANOVA analysis with the Dunnett’s multiple comparison test on log_10_-transformed values. Each bar represents the mean and standard error of 3 biological replicates. D-E) Stationary phase cultures of WT, *ΔyopB*, and Δ*dusB-fis* strains fused containing H-TEM or E-TEM beta-lactamase reporters were grown as in A-C. HEp-2 cells were infected at a multiplicity of infection of 10 for 1 hr and then incubated with CCF4 to determine the percentage of cells containing translocated effectors (% blue). Data was log_10_ transformed and statistical significance was calculated using One Way ANOVA analysis with Dunnett’s multiple comparison post-test comparing each strain to WT. Each bar represents the mean and standard error of 3–6 biological replicates. F-G) C57BL6/J mice were inoculated intravenously with a pool of 10^3^ bacteria, containing an equal mixture of *ΔdusB-fisΔyscF -*Kan^R^ and *ΔyscF*. 24 hours prior to and post-infection, mice were intraperitoneally injected with RB6-8C5 antibody. Mice were euthanized at 3 days post-infection and livers (F) and spleens (G) were collected, and dilutions of tissue homogenates were plated onto selective and non-selective media to determine the C.I. C.I. values were log_10_ transformed and statistical significance was calculated using a Mann-Whitney t-test. * indicates p≤0.05, ** indicates p≤0.01, **** indicates p≤0.0001.

Fis serves as a transcriptional regulator of virulence factors in several pathogens [34]. Therefore, we speculated that the virulence defect of Δ*dusB-fis* was due to an inability to mount a transcriptional response to protect against the bactericidal actions of neutrophils and inflammatory monocytes in systemic tissue sites. Neutrophils and inflammatory monocytes use a variety of mechanisms to restrict bacterial growth upon recruitment to tissue sites, including the phagocytosis of bacteria, release of toxic granules and diffusible reactive gases (ROS and reactive nitrogen species), and chelation of metals [35,36]. Because T3SS effectors interfere with many of these processes [37], and because Fis regulates expression of the SPI-1 and SPI-2 pathogenicity islands in *Salmonella* [38], we first tested whether Fis positively regulated expression of the T3SS or its effectors. Under conditions that induce expression and secretion of T3SS effectors, Δ*dusB-fis* and Δ*fis* mutants secreted effectors into culture supernatants at equivalent levels to WT *Yptb* (Fig 6A). Additionally, engineered strains of WT and Δ*dusB-fis Yptb* containing the beta-lactamase, TEM, fused to the first 100 amino acids of the T3SS effectors YopE or YopH [13,39] exhibited no difference in cleavage of the beta-lactamase substrate nitrocefin by those effectors (Fig 6B and 6C). Because Fis could regulate other factors, such as adhesins, which also contribute to efficient effector translocation into host cells [14], the ability of Δ*dusB-fis* to translocate T3SS effectors into cultured epithelial cells was measured using the CCF4-FRET based translocation assay. The Δ*dusB-fis* mutant had no defect in translocating YopE-TEM or YopH-TEM into cultured cells (Fig 6D and 6E), suggesting that Fis plays no role in regulating the expression of the *Yptb* T3SS machinery or in regulating the expression of other factors that promote efficient effector translocation through this system. Furthermore, a *dusB-fis* deletion generated in a strain lacking the T3SS needle was attenuated for virulence in the presence of Gr1^pos^ cells (Fig 6F and 6G), indicating that *dusB-fis* is critical for preventing growth restriction by phagocytes, even in the absence of a functional T3SS.

**Fig 7:**
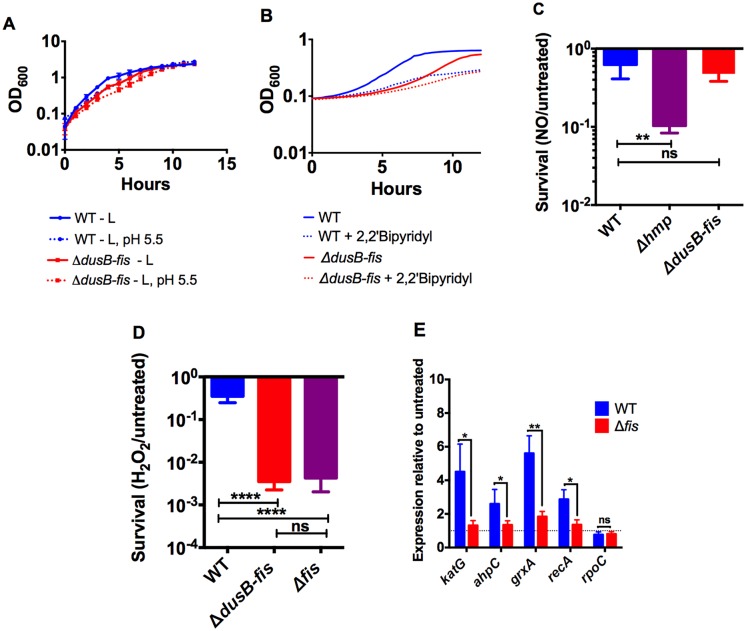
*dusB-fis* is required for resistance to oxidative stress. A) Stationary phase cultures of WT and *ΔdusB-fis* were diluted 1:100 into L broth and L broth adjusted to pH 5.5 and the OD_600_ of cultures was measured at 1-hour-intervals for 12 hours during growth with aeration. Each symbol represents the mean of 2–4 biological replicates. B) Stationary phase cultures were diluted 1:100 into a well of a 96-well plate containing L broth or L broth + 250 μM 2,2’- Bipyridyl and OD_600_ measurements were recorded at 15-minute intervals during growth with aeration. Lines represent the mean of 3 biological replicates. (C-D) Exponential phase cultures were washed and diluted 1:50 into M9 glucose medium or M9 glucose containing 2.5mM DETA NONOate (C) or M9 glucose containing 1.5mM H_2_O_2_ (D) for 60 minutes. Survival was calculated by determining the number of CFUs recovered following treatment divided by the number of CFUs recovered from untreated cultures. The mean and standard error of 3 biological replicates for DETA NONOate treatment and 6–10 biological replicates for H_2_O_2_ treatment are shown. Survival values were log_10_ transformed and statistical significance was calculated using One Way ANOVA analysis with Dunnett’s multiple comparison post-test comparing each strain to WT. E) *Δfis* fails to up-regulate ROS-responsive genes after exposure to H_2_O_2_. Exponential phase cultures were washed and diluted 1:50 into M9 glucose medium or M9 glucose containing 20 μM H_2_O_2_ and were incubated for 10 minutes with aeration. RNA isolated from treated and untreated samples was used to generate cDNA, and qPCR reactions were performed. Relative expression was determined by normalizing to 16S RNA as well as to expression in untreated samples using the ΔΔCT method. Bars represent the mean and standard error of 8 biological replicates. Unpaired Mann-Whitney t-tests were performed to calculate statistical differences between expression of each gene in WT and *Δfis*. * indicates p≤0.05, ** indicates p≤0.01, **** indicates p≤0.0001, ns indicates not significant.

To evaluate whether Fis promotes resistance to one or more of the bactericidal stresses imposed by neutrophils and inflammatory monocytes, the Δ*dusB-fis* mutant was exposed to several conditions that simulate the actions of these cells. These conditions included exposure to low pH (Fig 7A), low concentrations of iron (Fig 7B), nitric oxide (Fig 7C), and ROS (Fig 7D). While Δ*dusB-fis* was often delayed in entering into exponential growth compared to WT, its growth rate in broth with a low pH or titrated iron was not more impaired than a WT strain exposed to the same conditions (Fig 7A and 7B). Additionally, exposure to the nitric oxide donor DETA NONOate did not affect survival of Δ*dusB-fis*, but did result in killing of a mutant lacking *hmp*, which is known to play a role in nitric oxide detoxification by *Yptb* [15] (Fig 7C). By contrast, the survival of Δ*dusB-fis* and Δ*fis* strains was significantly impaired after exposure to H_2_O_2_ (Fig 7D), suggesting that Fis is required for resistance to ROS.

To determine whether Fis protects against ROS by altering the transcription of one or more genes that are responsive to ROS in other organisms [40–43], we performed qRT-PCR on transcripts isolated from WT and Δ*fis* following exposure to 20μM H_2_O_2_, a concentration that is sublethal to both strains (S5 Fig). In WT *Yptb*, these conditions were sufficient to induce transcription of four genes, *katG*, *ahpC*, *grxA*, and *recA* (Fig 7E). However, we observed significantly less transcriptional induction of these four genes in the Δ*fis* mutant (Fig 7E), suggesting that Fis promotes expression of these genes during oxidative stress. By contrast, there were no differences between WT and the Δ*fis* mutant in the expression of a non-ROS inducible gene, *rpoC*. In order to determine whether overexpression of a single ROS detoxifying protein was sufficient to restore growth of the Δ*fis* mutant after exposure to lethal concentrations of H_2_O_2_, the coding regions of *ahpC* and *katG*, which encode an alkyl hydroperoxide reductase and a catalase, respectively, and have been shown to contribute to H_2_O_2_ detoxification in other organisms [44–46], were each fused to a constitutive tetracycline promoter on the plasmid pACYC184 and introduced separately into WT and Δ*dusB-fis* strains. Notably, while expression of these genes was enhanced in the Δ*dusB-fis* mutant (S6A and S6B Fig), the sensitivities of these strains to H_2_O_2_ were no different from isogenic strains expressing *gfp* downstream of the same promoter (S6C Fig), indicating that expression of more than one Fis-regulated gene may be required to resist killing by H_2_O_2_. Alternatively, other regulatory targets, such as the SOS-response regulator *recA*, may play an essential role in Fis-dependent protection against oxidative stress. Combined, these results indicate that Fis protects against killing by ROS by either directly or indirectly regulating the transcription of multiple genes required for resistance to oxidative stress.

### Growth of Δ*dusB-fis* is restored in mice unable to generate ROS

**Fig 8:**
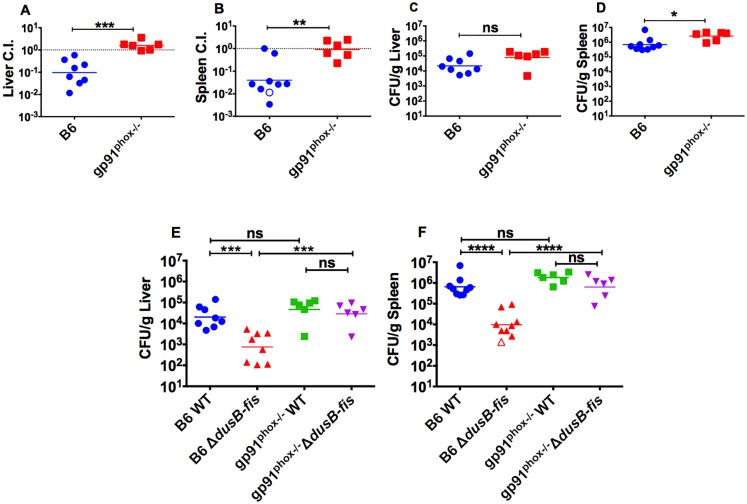
*ΔdusB-fis* is sensitive to ROS produced by the NADPH oxidase complex during mouse infection. C57Bl/6 or C57Bl/6 gp91^phox-/-^ mice were inoculated intravenously with 1x10^3^ CFU of a 1:1 mixture of WT and *ΔdusB-fis-*Kan^R^. Livers (A,C,E) and spleens (B,D,F) were collected, weighed, homogenized, and plated for CFUs on selective and non-selective agar at 3 days-post-infection. (A-B) The number of bacteria recovered from selective and non-selective plates was used to determine the C.I. of *ΔdusB-fis-*Kan^R^. Each data point represents an individual mouse. C.I. data was log_10_ transformed and statistical significance was calculated using the Mann-Whitney t-test. (C-D) The CFU/gram was determined by dividing the total number of recovered CFU on non-selective plates by the weight of the tissue. CFU/g data was log_10_ transformed and statistical significance was calculated using the Mann-Whitney t-test. (E-F) The number of bacteria recovered from selective and non-selective plates was used to determine the quantity of *ΔdusB-fis-*Kan^R^ and WT bacteria in each organ sample. CFU/g data was log_10_ transformed and statistical significance was calculated using One Way ANOVA analysis with the Dunnett’s multiple comparison test. * indicates p≤0.05, ** indicates p≤0.01, *** indicates 00.001, **** indicates p≤0.0001.

To test the possibility that Fis protects *Yptb* from ROS *in vivo*, gp91^phox-/-^ mice, which cannot assemble a productive NADPH oxidase complex [47], were infected with a mixture of WT and Δ*dusB-fis*. Strikingly, Δ*dusB-fis* was fully virulent in these mice (Fig 8A and 8B). Interestingly, gp91^phox-/-^ mice were only slightly more susceptible to *Yptb* infection, with total bacterial loads in the spleen and liver that were only ~3.5x higher than those in tissues recovered from C57Bl/6 mice (Fig 8C and 8D). Furthermore, most of this increase was attributed to the relief in Δ*dusB-fis* growth restriction in the gp91^phox-/-^ mice, as analysis of the CFU burden of each individual bacterial strain recovered from co-infected mice showed little difference in WT CFU levels between gp91^phox-/-^ and C57Bl/6 mice (Fig 8E and 8F). This result, coupled with our earlier observations, indicates that the primary role of *dusB-fis* during *Yptb* infection within deep tissue sites is to protect against ROS produced by neutrophils and inflammatory monocytes, likely by initiating a transcriptional response that enables *Yptb* to resist killing by ROS that have entered the bacterial cell.

## Discussion

As bacteria evolve to adapt and grow in different niches, they often acquire new traits through the acquisition of plasmids, pathogenicity islands, or integration of phages or other mobile genetic elements [48]. However, these organisms can also exploit or rely on other traits they previously possessed to survive in these new environments [48]. These previously held abilities likely reflect the environments that had been central to the survival of the organism in prior niches. Thus, the observation that pIB1^-^ strains of *Yptb* display a remarkable ability to grow and persist within several tissue sites during mammalian infection suggests that an ancestor of this bacterium may have relied on a number of chromosome-encoded factors to grow within mammalian tissue sites and withstand restriction by the host immune response prior to acquisition of the T3SS-encoding virulence plasmid. One of these host responses is the release of ROS, which are produced following the oxidative burst of phagocytic cells in response to fungal and bacterial infections in several tissue sites, including the GI tract, lungs, and systemic organs [49–51]. In phagocytic cells, oxidative burst occurs via the activation and assembly of the NADPH oxidase complex, usually in response to bacterial contact or pattern recognition receptor activation [52].

Our results, in aggregate, support a model (S7 Fig) whereby the *dusB-fis* operon in *Yptb* controls the transcription of genes critical for resisting killing by ROS that are generated by the NADPH oxidase complex of neutrophils and inflammatory monocytes surrounding bacteria. Specifically, (1) *dusB-fis* was important for defense against both neutrophils and inflammatory monocytes, as the growth of this mutant was only restored when both immune cell populations were depleted (Figs 3 and 4); (2) Δ*dusB-fis* initially colonized spleens and livers, but was unable to sustain growth in these tissue sites by 48 post-infection (Fig 5); (3) Fis was required for protection against oxidative stress (Fig 7D) and regulated the transcription of at least 4 genes, *katG*, *ahpC*, *grxA* and *recA*, which are predicted to contribute to resistance to ROS in *Yersinia* (Fig 7E)*;* and (4) growth of Δ*dusB-fis* was restored in gp91^phox-/-^ mice, whose immune cells lack a functional NADPH oxidase complex and thus cannot undergo oxidative burst (Fig 8). Remarkably, these mice contained equal bacterial loads of WT and Δ*dusB-fis* (Fig 8E and 8F), suggesting that the primary contribution of this operon to *Yptb* intravenous infection is to prevent against restriction by ROS.

The findings that *dusB-fis* was necessary for resisting killing by ROS *in vitro* and that this operon was dispensable for growth within gp91^phox91-/-^ mice were unanticipated for several reasons. First, previous studies of *Yersinia* gene expression in animal models have observed little transcriptional induction of ROS-detoxifying genes during infection of lymphoid tissue sites, suggesting that *Yersinia* may not experience oxidative stress during growth in these organs [15,53]. However, these studies were largely directed at analyzing the transcriptional induction of ROS-responsive genes compared to *in vitro* growth, where bacteria may also encounter some endogenous oxidative stress, and did not assess the survival of *Yptb* mutants lacking one or more of these genes during animal infection. In fact, a prior study analyzing the phenotype of a mutant lacking the superoxide dismutase *sodA* determined that this gene was critical for growth of *Y*. *enterocolitica* within livers and spleens, suggesting that *Yersinia* do encounter ROS during tissue infection [54]. Second, it has been well established that two T3SS effectors, YopE and YopH, prevent oxidative burst in Yop-intoxicated phagocytic cells [7,11]. However, YopE and YopH can only function within the cells into which they have been delivered. As only a small fraction of immune cells are intoxicated with Yops during infection of some tissue sites [13], it is possible that in these tissues, *Yptb* encounters ROS produced by non-injected cells, and would therefore require mechanisms to resist killing by these species. Because Fis is dispensable for T3SS effector translocation, but is required for protection against ROS both *in vivo* and *in vitro*, our work suggests that *Yptb* does encounter ROS during infection of the spleen and liver, and that these species must be coming from neighboring immune cells not intoxicated with Yops. Furthermore, the observation that gp91^phox-/-^ mice were no more sensitive to WT *Yptb* than C57Bl/6 mice (Fig 8E and 8F) suggests that WT *Yptb* is completely resistant to ROS produced by the immune response during infection. Therefore, we propose that *Yptb* utilizes both offensive and defensive measures to counteract ROS produced by phagocytic cells during mammalian infection by preventing oxidative burst in T3SS-intoxicated host cells, and also by initiating a Fis-dependent transcriptional response to protect against killing by ROS released by non-injected phagocytic cells (S7 Fig).

The *dusB-fis* operon is conserved in *Enterobacteriaceae* family members of the *Gammaproteobacteria* [55] and encodes the nucleoid-associated protein (NAP) Fis. While no published work has characterized a function for Fis in *Yersinia*, Fis and other NAPs have been well studied in *E*. *coli* and other organisms, where these small, histone-like proteins play important roles in modulating DNA architecture, as well as in directly and indirectly regulating transcription at a global level [34]. In *E*. *coli*, the two genes are co-regulated and transcribed from a single promoter upstream of *dusB* [56,57], where the *dusB* mRNA transcript is believed to play a regulatory role in promoting translation of Fis [55]. Interestingly, Fis serves as a transcriptional regulator of virulence factors in several mammalian pathogens, including *Vibrio cholerae*, *Shigella flexneri*, *Pasteurella multocida*, *Salmonella typhimurium*, and pathogenic *Escherichia coli* [34,38,58–65]. In these organisms, it activates a diverse range of virulence functions, including quorum sensing, capsule production, adhesion, and Type 3 Secretion [58–60,65]. Notably, a study performed in *E*. *coli* also characterized a role for Fis in protection against oxidative stress [66], suggesting that defense against ROS may be a conserved function of Fis across multiple bacterial species. However, the contribution of Fis to ROS resistance has not been examined in other pathogens.

Our findings indicate that, following exposure of *Yptb* to oxidative stress, Fis promotes the transcriptional induction of several ROS-detoxifying genes, as well the SOS response regulator *recA*. This suggests that Fis may prevent ROS-mediated killing of *Yptb* both by stimulating detoxification as well as by promoting the repair of DNA damage. Expressing the detoxifying genes *ahpC* and *katG* under the control of a constitutive promoter did not restore resistance of Δ*dusB-fis* to oxidative stress; however, this is not surprising because it is likely that Fis promotes expression of multiple genes that contribute to survival under these conditions. Future studies aimed at identifying global regulatory targets of this protein will further inform our understanding of how Fis promotes survival under these conditions.

Another surprise from our mini-TnSeq assay was the finding that the virulence defects of the *ΔpsaEFABC* and *ΔrfaH* mutants were exacerbated following neutrophil depletion during *Yptb* infections of the spleen and liver, respectively, indicating that these loci may promote survival in a non-inflammatory niche or in the presence of a host cell subset “unmasked” by neutrophil depletion. Consistent with this idea was the observation that the *ΔpsaEFABC* mutant was significantly attenuated in the pIB1^-^, but not the pIB1^+^, background in the spleen, as WT *Yptb* recruits a more robust, neutrophil-rich inflammatory response than its plasmid-deficient derivative in lymphoid tissue sites [16,17]. The *psaEFABC* operon encodes the fimbrial-like adhesin pH 6 antigen, which has been shown to contribute to lung colonization by *Y*. *pestis* [67]. Notably, *Y*. *pestis* undergoes an early “quiet” stage during infection by the pneumonic route, in which neutrophils are not recruited to the lungs until at least 24 hours post-infection [68]. *Y*. *pestis* may therefore require pH 6 antigen to colonize and grow within the lungs at early time-points because neutrophils have not yet been recruited. RfaH has been characterized as a global regulator of LPS synthesis in several gram-negative organisms, including *Y*. *enterocolitica*, where deletion of this gene results in a “rough” phenotype, in which core inner core LPS is exposed [69]. In *Y*. *pestis*, exposed core LPS promotes interactions with and uptake by dendritic cell subsets [70,71]. While neutrophils are the primary cells contacting bacteria during *Yptb* infection [15,17]**,** upon neutrophil depletion, it is possible that bacteria encounter dendritic cell subsets. Thus, during this condition, the *ΔrfaH* mutant may come into contact with and be phagocytosed by certain dendritic cell subsets. In contrast to the *ΔrfaH* and *ΔpsaEFABC* mutants, the virulence of a mutant lacking *YPK_3765* was restored in the absence of neutrophils, indicating that this gene is important for protection against clearance by these cells. *YPK_3765* is predicted to encode a murein peptide ligase (Mpl), a class of proteins important for peptidoglycan synthesis and recycling in other organisms [72]. Peptidoglycan is a known activator of pattern recognition receptors [73], so a loss of this protein in *Yptb* may result in aberrant expression or release of peptidoglycan outside of the bacterial cell, which could further enhance killing by neutrophils during tissue infection.

Our mini-TnSeq assay offers a number of advantages in evaluating smaller cohorts of mutants initially identified in a large transposon-based screen, where extreme bottlenecks can inhibit the ability of an otherwise competent mutant to colonize tissues. In addition, transposon disruptions of genes can often have polar effects on the expression of nearby loci. Finally, screening individual mutants in single-strain and 1:1 co-infections often requires large numbers of mice. To address these issues, our assay utilizes deep sequencing as a read-out, where in-frame mutants contain scar sequences that can be used as a primer template for PCR amplification of Illumina libraries. This allowed us to use small pools of bacterial mutants in mouse infections, thereby bypassing bottleneck issues and also minimizing animal usage. Unlike our initial study, in which the operon containing the most significantly attenuated transposon mutant, *mrtAB*, was not required for systemic infection by a pIB1^+^ strain [17], the vast majority of mutants with defects in the absence of the virulence plasmid were also attenuated for infection of pIB1^+^ *Yptb* in the liver. In fact, only one mutant, Δ*oppD*, was attenuated for growth in livers only in the absence of pIB1. Curiously, both *oppD* and *mrtAB* encode components of transporters, suggesting that they could carry out functions that are redundant with the T3SS, or that they are critical for survival in niches that are not predominantly inhabited by WT *Yptb*.

Interestingly, six mutants, Δ*aroA*, Δ*YPK_3184*, Δ*arnDT*, Δ*YPK_1920*, Δ*YPK_2594*, and Δ*psaEFABC*, were defective for growth of the WT strain in the liver, but not the spleen, reflecting the fact that the original screen was performed in the liver and suggesting that different tissue sites can influence the repertoire of bacterial virulence factors required during infection. Indeed, it has been established that mammalian organs differ in their mechanisms of sensing and responding to microbial infections [74,75] and that, consequently, bacteria may utilize genes to survive in some tissues that are dispensable in others. For example, the bacterial pathogen *Francisella tularensis* specifically requires tryptophan biosynthesis genes during infection of the lung in order to counteract restriction of this amino acid by a host-encoded enzyme expressed in this organ [76]. Likewise, *Yptb* may require certain factors, such as *aroA*, to survive in the liver because their products are limiting in this organ. Additionally, certain mutants, such as Δ*YPK_3184* and Δ*arnDT*, may be more readily detected by pattern recognition receptors in the liver and would therefore fail to colonize or sustain growth in this organ. Future work with these mutants may help to uncover host immune mechanisms specific to this tissue site.

Altogether, our findings reinforce the argument that *Yptb* relies on a number of chromosome-encoded defense factors to grow within tissue sites and withstand restriction by immune cells. In particular, the small, histone-like protein Fis plays a critical role in protecting *Yptb* from ROS produced by phagocytic cells during tissue infection. Future work will be aimed at identifying the global network of Fis- regulated genes during conditions of oxidative stress to understand how this protein promotes bacterial adaptation to this condition.

## Materials and Methods

### Ethics statement

This study was performed in accordance with the recommendations in the Guide for Care and Use of Laboratory Animals of the National Institutes of Health. The Institutional Animal Care and Use Committee (IACUC) of Tufts University approved all animal procedures. Our approved protocol numbers were B2012-54 and B2015-35. All efforts were made to minimize suffering; animals were monitored following infection and were euthanized upon exhibiting substantial signs of morbidity by CO_2_ asphyxiation followed by cervical dislocation.

### Bacterial strain construction

Strains utilized in this study are listed in S1 Table and primers are listed in S2 Table. *Yptb* gene deletions were generated in pIB1^-^ YPIII and pIB1^+^ IP2666, as indicated in S1 Table. Deletions replacing genes of interest with in-frame scar sequences were created using allelic exchange as follows: primers were designed to amplify ~800bp regions directly up and downstream of each targeted gene (S2 Table). These oligos also contained overlapping sequences necessary to create a ~60bp scar sequence after gene deletion. Overlapping products were combined using splicing by overlap extension (SOE) PCR and ligated into the *sacB-*based vector pCVD442 following restriction digestion. The resulting plasmids were introduced into *E*.*coli* DH5αλpir and integrated into the *Yptb* chromosome by mating in the presence of a third mating strain containing pRK600. Deletions were confirmed by PCR utilizing primers located 800bp up and downstream of the deleted gene. For *fis* deletion, primers with overlapping sequences were designed to amplify ~800bp regions directly up and downstream of the gene. These products were combined by SOE PCR, ligated into pCVD442, and the resulting plasmid was introduced into *E*. *coli* DH5αλpir and mated into *Yptb* as described above. Deletion of *fis* was confirmed by PCR. To complement *dusB-fis*, the entire operon and 800bp of both up and downstream sequences was amplified, and the product was cloned into pCVD442 by restriction digestion and ligation. The resulting plasmid was introduced into *E*. *coli* DH5αλpir and mated into *Yptb ΔdusB-fis* and successful restoration of the operon was confirmed by PCR. Strains containing YopE-TEM and YopH-TEM fusions were generated by mating *Yptb* strains with a SM10λpir strain containing the plasmid pSR47-YopETEM or pSR47-YopHTEM [13,39]. Following conjugation, bacteria were plated on kanamycin and irgasan to select for crossover of the chimeric YopE- or Yop-HTEM genes into the *yopE* and *yopH* loci. Successful crossover was confirmed by PCR. To generate strains constitutively expressing either *ahpC* or *katG*, the open reading frames of these genes were amplified by PCR and products were fused downstream of a constitutive tetracycline promoter on the plasmid pACYC184-*gfp* [17], using PCR [77] to replace the open reading frame of *gfp* with each respective product. Successful integration of *ahpC* and *katG* open reading frames was confirmed by sequencing, and plasmids were introduced into WT and *ΔdusB-fis* strains by electroporation and selection with 20mg/mL chloramphenicol.

### Media and growth conditions

All *Yptb* cultures were grown in L broth, with the exception of nitric oxide and H_2_O_2_ sensitivity assays (described below). Following mouse infections, tissue homogenates were plated onto L agar containing 0.5 μg/mL irgasan or a combination of 0.5 μg/mL irgasan and 50 μg/mL kanamycin to select for marked bacterial strains. During strain construction, 50 μg/mL carbenicillin and 0.5 μg/mL irgasan were used to select for strains containing integrated plasmids following matings, and 10% sucrose was utilized to select for strains that had resolved the integrated plasmid. With the exception of the T3SS and translocation assays, all cultures were incubated at 26°C with aeration. For animal infections, strains were inoculated into L broth 48 hours prior to infection. Following overnight growth, these strains were diluted 1:40 and incubated for ~8 hours, after which they were diluted 1:100 and incubated overnight.

### Mouse infections and immune cell depletions

All infections were performed by intravenous injection in 8–10 week C57Bl/6 or C67Bl/6 gp91^phox-/-^ mice obtained from Jackson, NCI, and Taconic labs. For infections with strains constructed in pIB1^-^ YPIII, mice were inoculated with 1 x 10^4^ bacteria. For infections with strains constructed in pIB1^+^ IP2666, mice were inoculated with 1 x 10^3^ bacteria. Competition experiments were performed using a 1:1 mixture of an unmarked strain and a strain harboring an insertion of miniTn5 Kan^R^ in a neutral locus [78]. Following infections, spleens and livers were isolated, weighed, homogenized, and plated on L agar containing 0.5 μg/mL irgasan. The quantity of CFU/gram of organ was determined by dividing the number of recovered CFU by the weight of the tissue sample extracted, or in cases where the entire organ was extracted, CFU/organ values were determined. For competition experiments, tissue homogenates were plated onto non-selective media as well as onto media containing 50 μg/mL kanamycin. The CFU count for each strain was determined by subtracting the number of Kan^R^ colonies from the total number of colonies recovered on non-selective plates. The proportion of each strain in the inoculum was confirmed using the same methods. C.I values were determined by the following equation: C.I. = (mutant/WT output ratio)/(mutant/WT input ratio). For Ly6G and Gr1 cell depletions, mice were intraperitoneally injected with 50 μg of 1A8 (Fisher) or RB6-8C5 (eBioscience) antibody 24 hours prior to and 24 hours post-infection. For inflammatory monocyte depletions, mice were intraperitoneally injected with 20 μg of MC-21 antibody [79] 1 day prior to infection and each day after until completion of the experiment. To confirm successful neutrophil and inflammatory monocyte depletion, spleen homogenates were stained with CD11b PE-Cy7 (eBioscience) and Gr1 PE Cy-5 (eBioscience) and analyzed by flow cytometry, as previously described [80].

### Mini-TnSeq infections and analysis

Overnight cultures of individual strains were mixed so that each putatively attenuated mutant would represent ~3% of the inoculum, and the combined neutral mutants would represent ~50% of the inoculum. Libraries were intravenously injected into 10 untreated C57Bl/6 mice and 7–8 C57Bl/6 mice treated with either RB6-8C5 or with 1A8. At 3 days post-infection, tissues were isolated, homogenized, and plated for CFUs on 150mm agar plates so that each plate would contain ~1x10^4^ CFUs. Bacteria were scraped off plates, mixed, and genomic DNA was extracted from a volume equivalent to ~2x10^9^ CFUs using the Qiagen DNeasy Blood and Tissue kit. DNA libraries were prepared for sequencing using the homopolymer tail-mediated ligation PCR technique as previously described [81]. Briefly, genomic DNA was sheared by sonication and treated with terminal deoxytransferase in order to generate a 3’ poly C-tail sequence. Two rounds of nested PCR were then employed to amplify regions immediately downstream of deleted genes. These products were multiplexed using 6bp indexing primers and sequenced on the Illumina Hi-Seq 2500. Following sequencing, reads were mapped to the region immediately downstream of the deleted genes and the total number of reads for each mutant in a given organ or input pool was divided by the total number of reads obtained for that organ or input pool. Fitness values were obtained by dividing the abundance of a mutant in a given organ by its abundance in the input pool.

### T3SS secreted protein assay

Strains were grown overnight at 26°C with aeration, then diluted 1:40 into L broth containing 20mM sodium oxalate + 20mM MgCl_2_. Cultures were grown for 2 hours at 26°C with aeration and then shifted to 37°C for 2 hours and grown with aeration. Following growth, the OD_600_ of each culture was measured and strains were diluted to achieve equivalent optical densities. Cultures were centrifuged and 10% trichloroacetic acid was added to culture supernatants to precipitate all secreted proteins. Precipitated proteins were pelleted by centrifugation, washed with acetone, and resolved by electrophoresis on a 12.5% SDS-polyacrylamide gel.

### Beta-lactamase assays

Strains containing chimeric YopE-TEM and YopH-TEM fusions were grown overnight at 26°C with aeration, then diluted 1:40 into L broth containing 20mM sodium oxalate + 20mM MgCl_2_. Cultures were grown for 2 hours at 26°C with aeration and then shifted to 37°C for 2 hours and grown with aeration. When performing T3SS assays, the OD_600_ of each culture was measured and strains were diluted to achieve equivalent optical densities. Cultures were centrifuged and 40 μL of the culture supernatant was applied to 10 μL of 500 μg/mL nitrocefin, for a final concentration of 100 μg/mL. After a 10-minute incubation, the A_490_ of samples was measured using a BioTek Synergy HT plate reader. When performing translocation assays, cultures were used to infect HEp-2 cells at the indicated multiplicities of infection. After 1 hour, cells were treated with gentamicin to stop the infection. Cells were lifted from plates using trypsin and then treated with 1 μg/ml CCF4 (Invitrogen) and 1.5 mM probenecid (Sigma). Following a 20-minute incubation, cells were analyzed by flow cytometry to quantify fluorescence following excitation at a 388 nm and blue fluorescence (450nm) and green fluorescence (530) were measured. Blue fluorescence indicated the presence of translocated effectors inside of the cell. The %blue cells were determined by dividing the number of blue cells by the total number of cells analyzed in a given sample.

### Growth in low pH and low iron

For low pH growth assays, WT and *ΔdusB-fis Yptb* were grown overnight at 26°C with aeration, then diluted 1:100 into either L broth or L broth at pH 5.5. Cultures were grown at 26°C with aeration, and the OD_600_ of cultures was measured at 1-hour intervals for 12 hours. For low iron growth assays, cultures were grown overnight as described above and diluted 1:100 into a well of a 96-well plate containing L broth or L broth containing 250 μM 2,2’- Bipyridyl (Sigma). Plates were incubated for 20 hours in a BioTek Synergy HT plate reader at 26°C with aeration, and OD_600_ measurements were recorded for each well at 15-minute intervals.

### Nitric oxide and H_2_O_2_ sensitivity assays

Stationary phase cultures were diluted 1:40 into L broth and grown for 4 hours at 26°C with aeration. Cultures were then washed and diluted 1:50 into M9 glucose medium or into M9 glucose medium containing either 1.5mM H_2_O_2_ or 2.5mM of the nitric oxide donor DETA NONOate (Cayman Chemical). Samples were incubated at 26°C with aeration for 1 hour, and dilutions were then plated onto L agar in order to quantify surviving bacteria.

### qRT-PCR

Stationary phase cultures were diluted 1:40 into L broth and grown for 4 hours at 26°C with aeration. Cultures were then washed and diluted 1:50 into M9 glucose medium or into M9 glucose medium containing 20 μM H_2_O_2_, and were incubated with aeration for 10 minutes. For experiments with strains containing pACYC184-*ptet*::*katG* and pACYC184-*ptet*::*ahpC*, cultures were incubated with 1.5mM H_2_O_2_ for 60 minutes prior to RNA isolation. H_2_O_2_ -treated samples were pelleted and resuspended in buffer RLT (Qiagen) + ß-mercaptoethanol, and RNA was isolated using the Qiagen RNeasy kit. DNA contamination was eliminated using the DNA-free kit (Ambion), and RNA was reverse transcribed into cDNA using M-MLV reverse transcriptase (Invitrogen), in the presence of RNase-OUT (Invitrogen). cDNA was utilized as a template in qPCR reactions with 0.5μM F and R primers (S2 Table) and SYBR Green (Applied Biosystems), using the BioRad CFX Real-Time PCR detection system. Samples were normalized to an endogenous 16S RNA control and relative expression was determined using the ΔCT and ΔΔCT methods (Applied Biosystems), when comparing treated to untreated samples.

### Accession numbers

Accession numbers for the genes described in this study in NCBI are: *aroA*, YPK_2670; *aroE*, YPK_0321; *purM*, YPK_1253; *rfaH*, YPK_3937; *wecC*, YPK_4030; *arnDT*, YPK_1834-YPK_1835; *dusB*, YPK_0453; *fis*, YPK_0452; *flgD*, YPK_2423; *psaEFABC*, YPK_2761-YPK_2757; *katG*, YPK_3388; *ahpC*, YPK_3267; *grxA*, YPK_2733; *recA*, YPK_3375; *rpoC*, YPK_0341.

## Supporting Information

S1 Fig Mini-TnSeq assay schematic. (A) Mutants containing identical in-frame scar sequences were prepared for Illumina sequencing. Briefly, genomic DNA was isolated from bacterial input and output pools, sheared by sonication, and treated with terminal deoxytransferase in order to generate a 3’ poly C-tail sequence. Two rounds of nested PCR were then employed to amplify regions immediately downstream of deleted genes. These products were multiplexed using 6bp indexing primers and sequenced on the Illumina Hi-Seq 2500. (B) Depiction of mini-TnSeq experiment. Infection inoculums were prepared so that each putatively attenuated mutant represented ~3% of the pool, and the combined neutral strains represented 50%. Mice were infected with pIB1^+^ and pIB1^-^ libraries, and after 3 days, spleens and livers were isolated, homogenized, and plated to retrieve surviving bacteria. DNA was prepared for sequencing as indicated in (A) and fitness values were calculated by determining the percentage of reads for a mutant in an organ by the percentage of that mutant in its respective input pool.
(TIFF)

S2 Fig Bacterial loads following infections of mini-TnSeq libraries. Bacterial loads recovered from (A) spleens and (B) livers at 3-days post-infection of mini-TnSeq libraries. A dose of 10^4^ CFUs was administered for YPIII/pIB1^-^ libraries and a dose of 10^3^ CFUs was administered for IP2666/pIB1^+^ libraries. Each data point represents an individual mouse. N = 8–18 mice. CFU values were log_10_ transformed and statistical significance was calculated using a Mann-Whitney t-test. *** indicates p≤0.001; ns indicates not significant.
(TIFF)

S3 Fig Virulence of mini-TnSeq library in depleted mice. Fitness of mutants in mini-TnSeq library following depletion of Gr1^pos^ or Ly6G^pos^ cells. Mice were intraperitoneally injected with RB6-8C5 (A-B) or 1A8 (C-D) 24 hours prior to and post-infection. Mice were inoculated intravenously with libraries of knockouts generated in IP2666/pIB1^+^ at a dose of 10^3^ CFU. Fitness values were obtained by dividing the proportion of sequencing reads for a mutant in the depleted liver (A, C) or spleen (B,D) by its proportion of reads in the inoculum. Each data point for a mutant represents an individual mouse. N = 4–10 mice. Non-depleted fitness values are the same as reported in Fig 1. Fitness scores values were log_10_ transformed and an unpaired t-test with the Holm-Sidak correction for multiple comparisons was performed to calculate statistical differences between the fitness scores of specific bacterial mutants in depleted versus non-depleted mice. * indicates p≤0.05, ** indicates p≤0.01, *** indicates p≤0.001, and **** indicates p≤0.0001.
(TIFF)

S4 Fig Confirmation of neutrophil and inflammatory monocyte depletions. (A) Representative FACs plots from spleens isolated from infected mice that were not depleted, or mice treated with either 1A8 or MC-21 antibodies. Tissues were extracted 3 days post-infection and single cell suspensions were prepared for FACs analysis by staining with Gr1 and Cd11b antibodies. Gating was performed as indicated, where neutrophils were designated as those cells expressing high levels of both Gr1 and Cd11b, and inflammatory monocytes were designated as those cells expressing intermediate levels of Gr1 and high levels of Cd11b. (B) Quantitation of FACs analysis described above. Statistical significance was determined using One Way ANOVA analysis with Dunnett’s multiple comparison post-test comparing the % total cell values of each depletion condition with that of non-depleted mice. * indicates p≤0.05 and ** indicates p≤0.01.
(TIFF)

S5 Fig Sensitivity of Δ*fis* to sub-millimolar concentrations of H_2_O_2_. Exponential phase cultures were washed and diluted 1:50 into M9 glucose medium or M9 glucose containing the indicated concentrations of H_2_O_2_ and incubated with aeration for 10 minutes. Survival was calculated by determining the number of CFUs recovered following treatment divided by the number of CFUs recovered from untreated cultures. The mean and standard error of 2 biological replicates (for 50 and 100 μM treatments), 4 biological replicates (for 10 μM treatment) or 8 biological replicates for (for 20 μM treatment) are shown.
(TIFF)

S6 Fig Constitutive expression of *katG* or *ahpC* does not restore resistance of *dusB-fis* to ROS. Exponential phase cultures were washed and diluted 1:50 into M9 glucose medium or M9 glucose containing 1.5mM H_2_O_2_ and incubated with aeration for 60 minutes. (A-B) Following treatment, RNA was isolated from samples exposed to H_2_O_2_, which was used to generate cDNA, and qPCR reactions were performed. Relative expression was determined by normalizing to 16S RNA using the ΔCT method. Bars represent the mean and standard error of 3–7 biological replicates. (C) A fraction of each H_2_O_2_-treated and untreated culture was also plated to determine surviving bacteria. Survival was calculated by determining the number of CFUs recovered following treatment divided by the number of CFUs recovered from untreated cultures.
(TIFF)

S7 Fig Proposed model of *Yptb* resistance to ROS during growth within systemic tissue sites. During growth within livers and spleens, *Yptb* forms extracellular aggregates or microcolonies [15,16]. Following stimulation of the immune response by infecting bacteria, neutrophils, macrophages, and inflammatory monocytes are recruited to sites of infection. (A) Some cells in close contact with bacteria on the periphery of the microcolony become translocated with T3SS effectors, (B) resulting in inhibition of NADPH oxidase activation and oxidative burst. (C) Other cells, which are not translocated with Yops, undergo oxidative burst in response to bacterial contact and/or PRR activation. (D) In WT infections, ROS released by these cells diffuse into the *Yptb* microcolony, where their bactericidal effects are resisted in a Fis-dependent manner, potentially through transcriptional induction of ROS-responsive genes. However, in Δ*fis* infections, mutants are unable to resist the bactericidal effects of ROS and are killed or restricted for growth.
(TIFF)

S1 Table Strains and plasmids. List of strains and plasmids that were constructed and/or utilized in this study.
(DOCX)

S2 Table List of primers. List of primers utilized in this study for generation of pCVD442 and pACYC184 plasmids, preparation and sequencing of Illumina libraries, and qRT-PCR analysis.
(DOCX)
