# Supplementary figures and images for: Fis Is Essential for Yersinia pseudotuberculosis Virulence and Protects against Reactive Oxygen Species Produced by Phagocytic Cells during Infection
Source: PLoS Pathog. 2016 Sep 30;12(9):e1005898. doi: 10.1371/journal.ppat.1005898 (PMC5045184; doi:10.1371/journal.ppat.1005898)

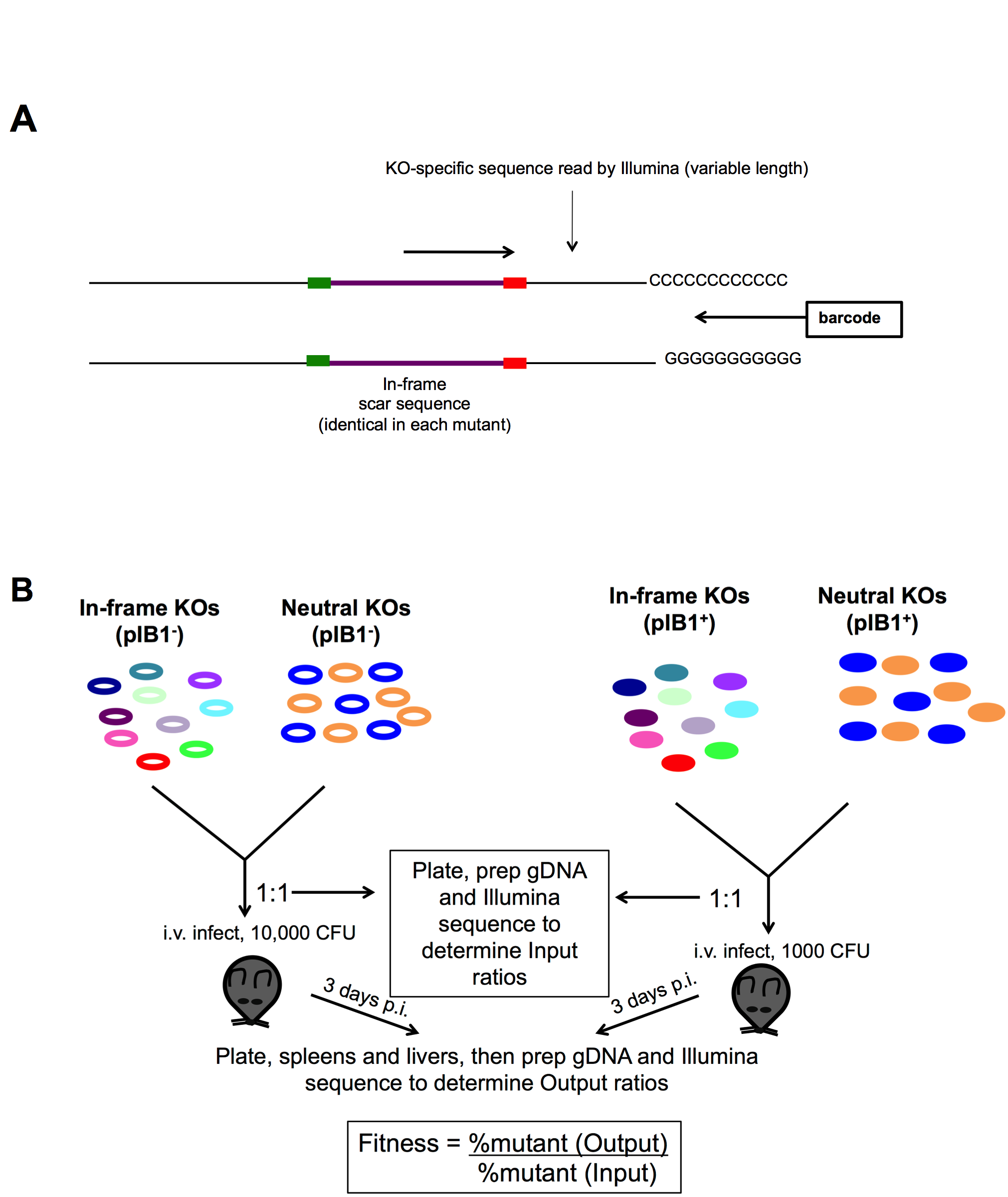

Supplement: S1 Fig — (A) Mutants containing identical in-frame scar sequences were prepared for Illumina sequencing. Briefly, genomic DNA was isolated from bacterial input and output pools, sheared by sonication, and treated with terminal deoxytransferase in order to generate a 3’ poly C-tail sequence. Two rounds of nested PCR were then employed to amplify regions immediately downstream of deleted genes. These products were multiplexed using 6bp indexing primers and sequenced on the Illumina Hi-Seq 2500. (B) Depiction of mini-TnSeq experiment. Infection inoculums were prepared so that each putatively attenuated mutant represented ~3% of the pool, and the combined neutral strains represented 50%. Mice were infected with pIB1+ and pIB1- libraries, and after 3 days, spleens and livers were isolated, homogenized, and plated to retrieve surviving bacteria. DNA was prepared for sequencing as indicated in (A) and fitness values were calculated by determining the percentage of reads for a mutant in an organ by the percentage of that mutant in its respective input pool. (TIFF) [file ppat.1005898.s001.tiff]

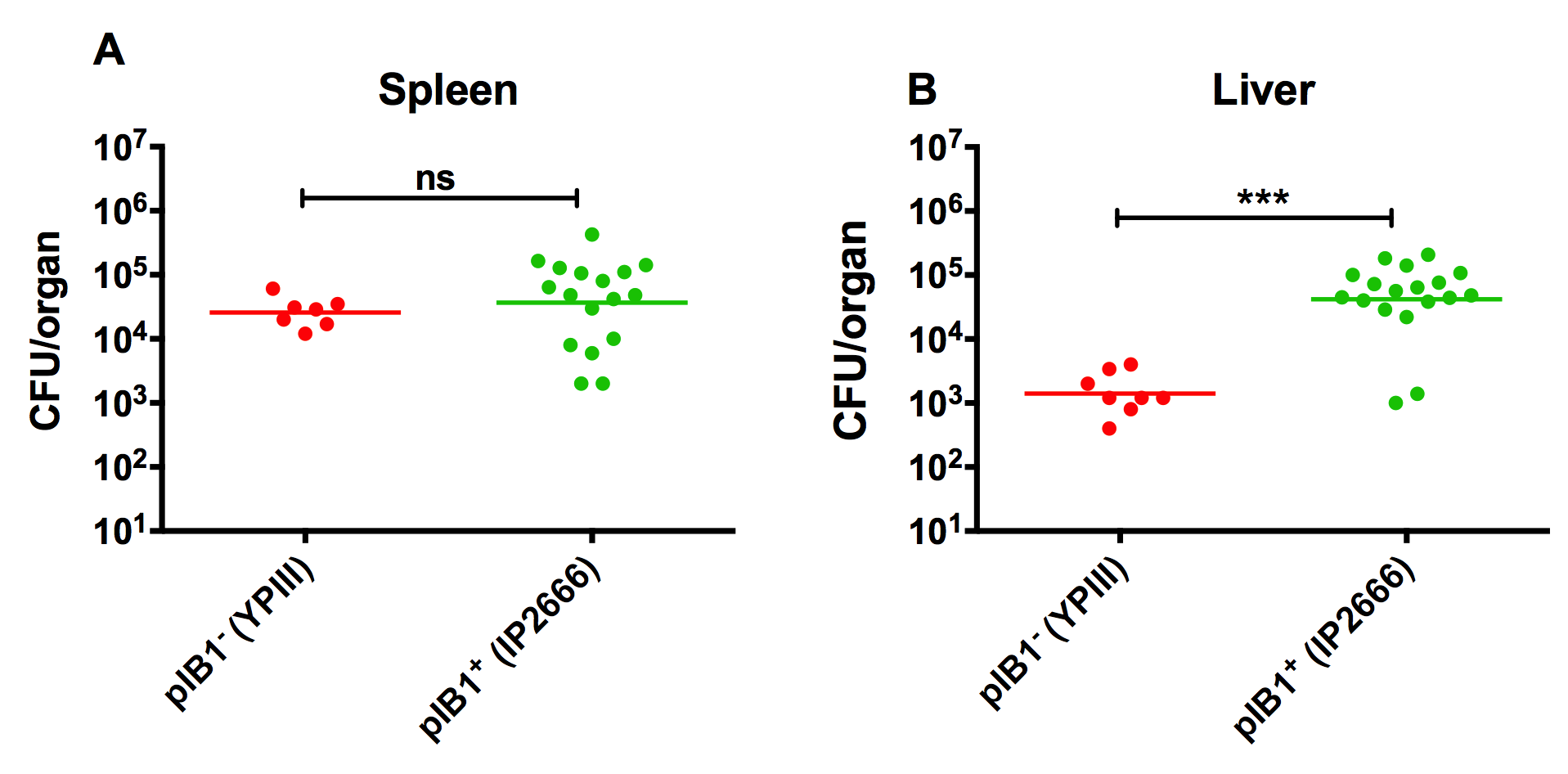

Supplement: S2 Fig — Bacterial loads recovered from (A) spleens and (B) livers at 3-days post-infection of mini-TnSeq libraries. A dose of 104 CFUs was administered for YPIII/pIB1- libraries and a dose of 103 CFUs was administered for IP2666/pIB1+ libraries. Each data point represents an individual mouse. N = 8–18 mice. CFU values were log10 transformed and statistical significance was calculated using a Mann-Whitney t-test. *** indicates p≤0.001; ns indicates not significant. (TIFF) [file ppat.1005898.s002.tiff]

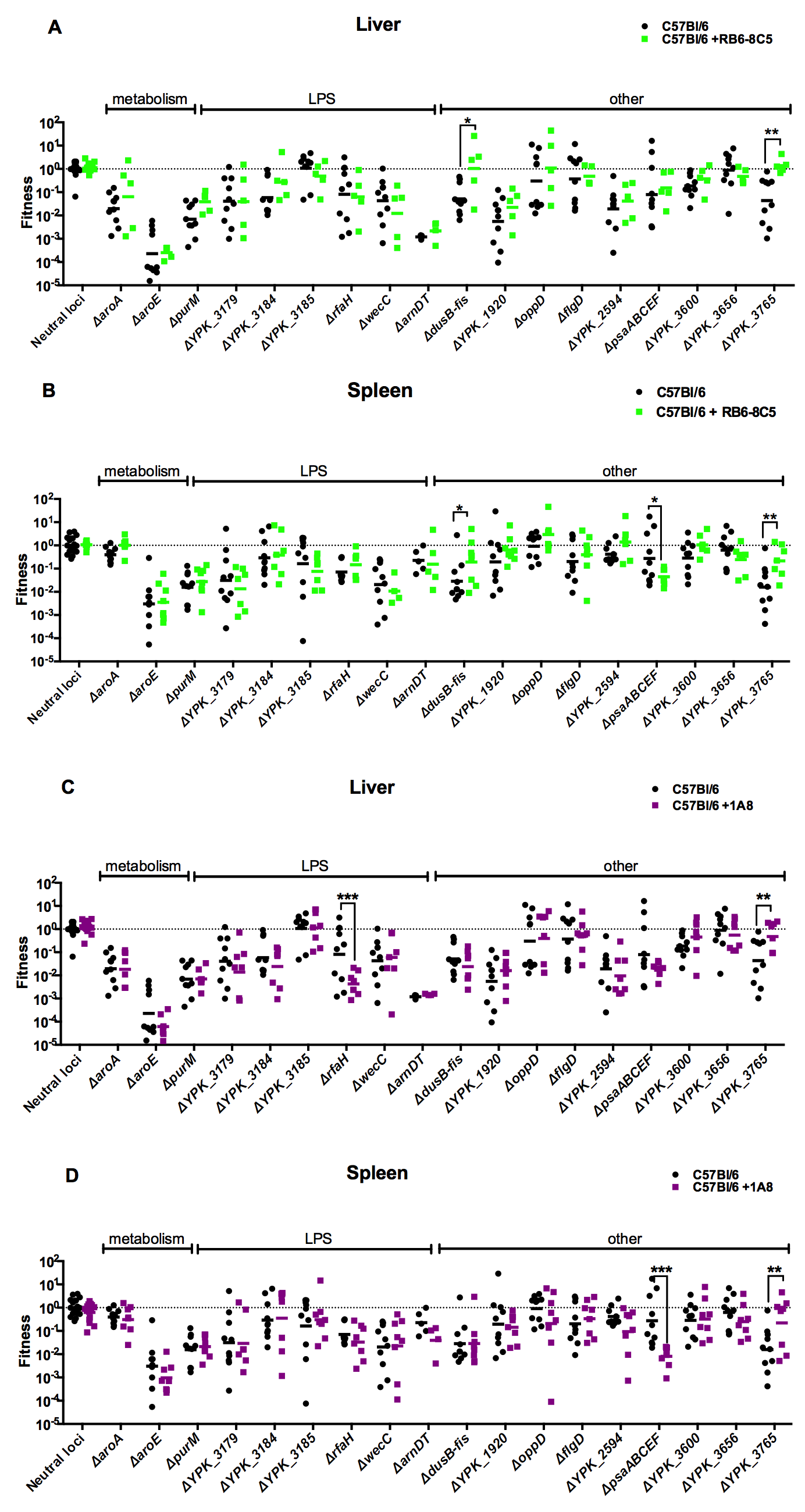

Supplement: S3 Fig — Fitness of mutants in mini-TnSeq library following depletion of Gr1pos or Ly6Gpos cells. Mice were intraperitoneally injected with RB6-8C5 (A-B) or 1A8 (C-D) 24 hours prior to and post-infection. Mice were inoculated intravenously with libraries of knockouts generated in IP2666/pIB1+ at a dose of 103 CFU. Fitness values were obtained by dividing the proportion of sequencing reads for a mutant in the depleted liver (A, C) or spleen (B,D) by its proportion of reads in the inoculum. Each data point for a mutant represents an individual mouse. N = 4–10 mice. Non-depleted fitness values are the same as reported in Fig 1. Fitness scores values were log10 transformed and an unpaired t-test with the Holm-Sidak correction for multiple comparisons was performed to calculate statistical differences between the fitness scores of specific bacterial mutants in depleted versus non-depleted mice. * indicates p≤0.05, ** indicates p≤0.01, *** indicates p≤0.001, and **** indicates p≤0.0001. (TIFF) [file ppat.1005898.s003.tiff]

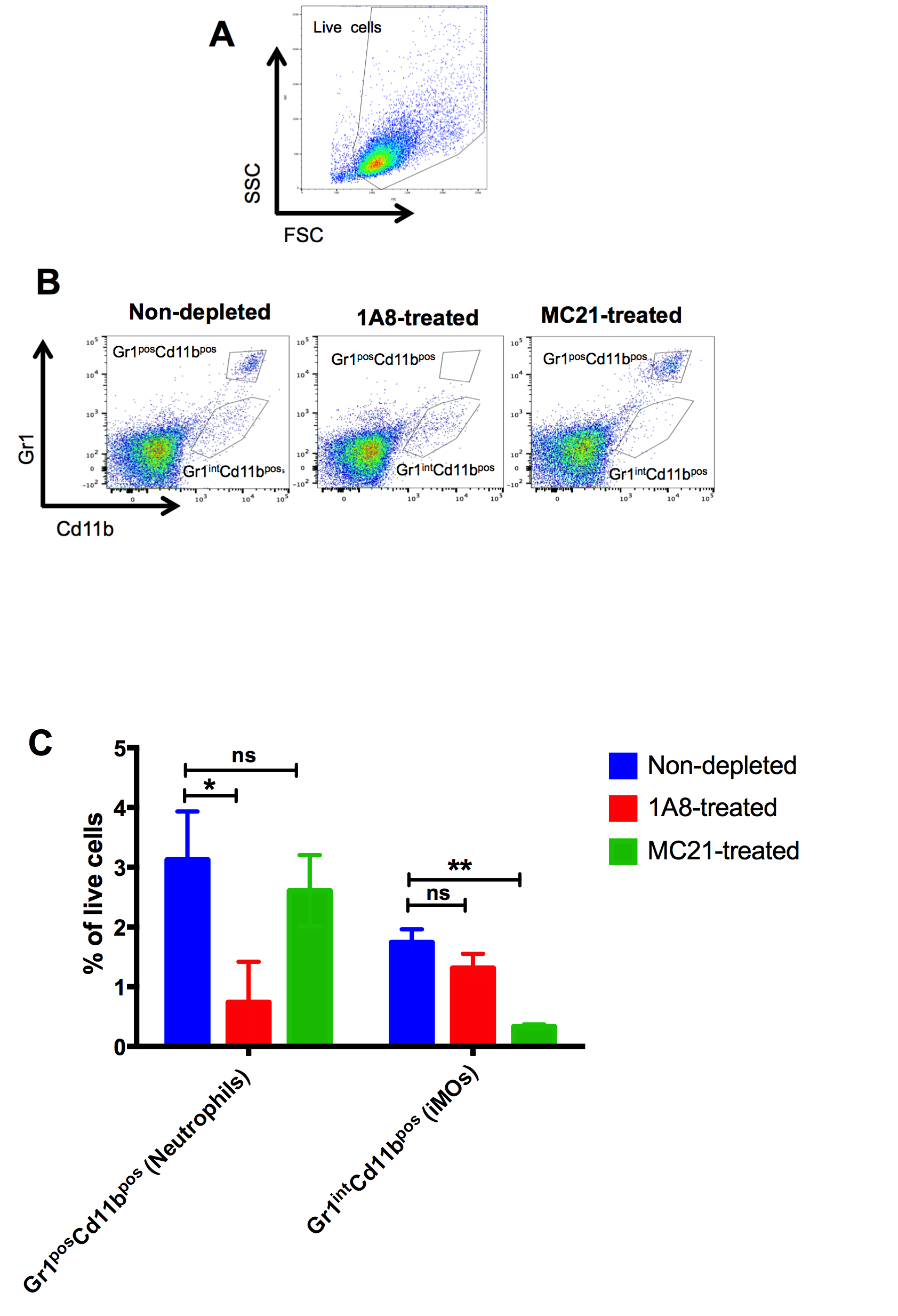

Supplement: S4 Fig — (A) Representative FACs plots from spleens isolated from infected mice that were not depleted, or mice treated with either 1A8 or MC-21 antibodies. Tissues were extracted 3 days post-infection and single cell suspensions were prepared for FACs analysis by staining with Gr1 and Cd11b antibodies. Gating was performed as indicated, where neutrophils were designated as those cells expressing high levels of both Gr1 and Cd11b, and inflammatory monocytes were designated as those cells expressing intermediate levels of Gr1 and high levels of Cd11b. (B) Quantitation of FACs analysis described above. Statistical significance was determined using One Way ANOVA analysis with Dunnett’s multiple comparison post-test comparing the % total cell values of each depletion condition with that of non-depleted mice. * indicates p≤0.05 and ** indicates p≤0.01. (TIFF) [file ppat.1005898.s004.tiff]

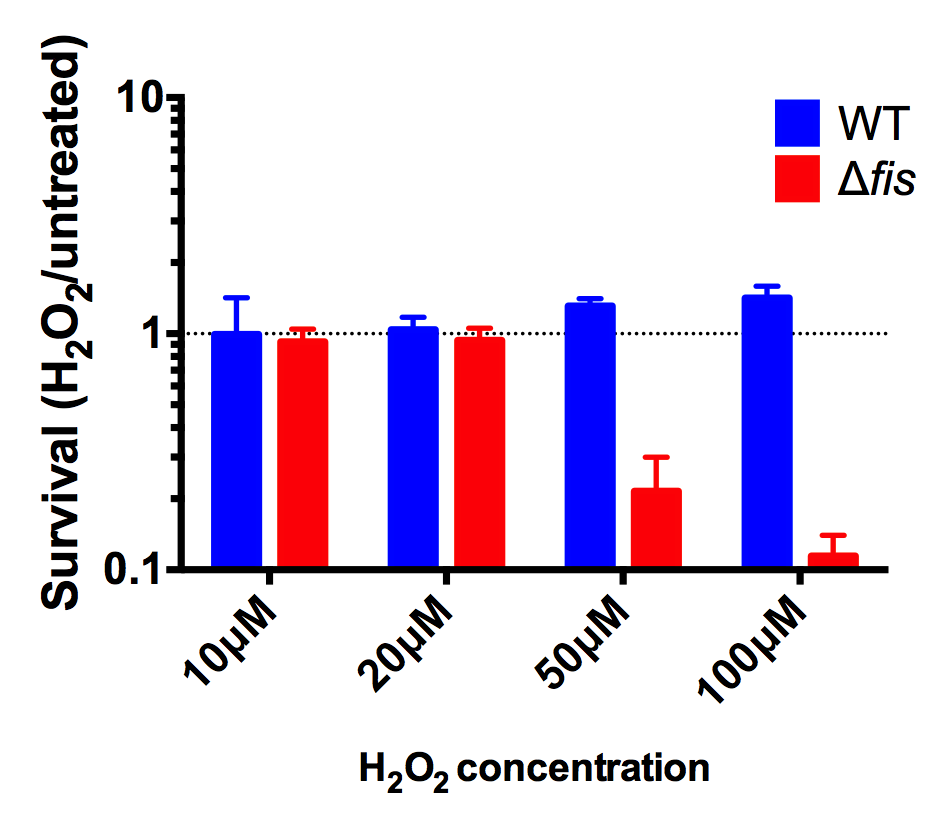

Supplement: S5 Fig — Exponential phase cultures were washed and diluted 1:50 into M9 glucose medium or M9 glucose containing the indicated concentrations of H2O2 and incubated with aeration for 10 minutes. Survival was calculated by determining the number of CFUs recovered following treatment divided by the number of CFUs recovered from untreated cultures. The mean and standard error of 2 biological replicates (for 50 and 100 μM treatments), 4 biological replicates (for 10 μM treatment) or 8 biological replicates for (for 20 μM treatment) are shown. (TIFF) [file ppat.1005898.s005.tiff]

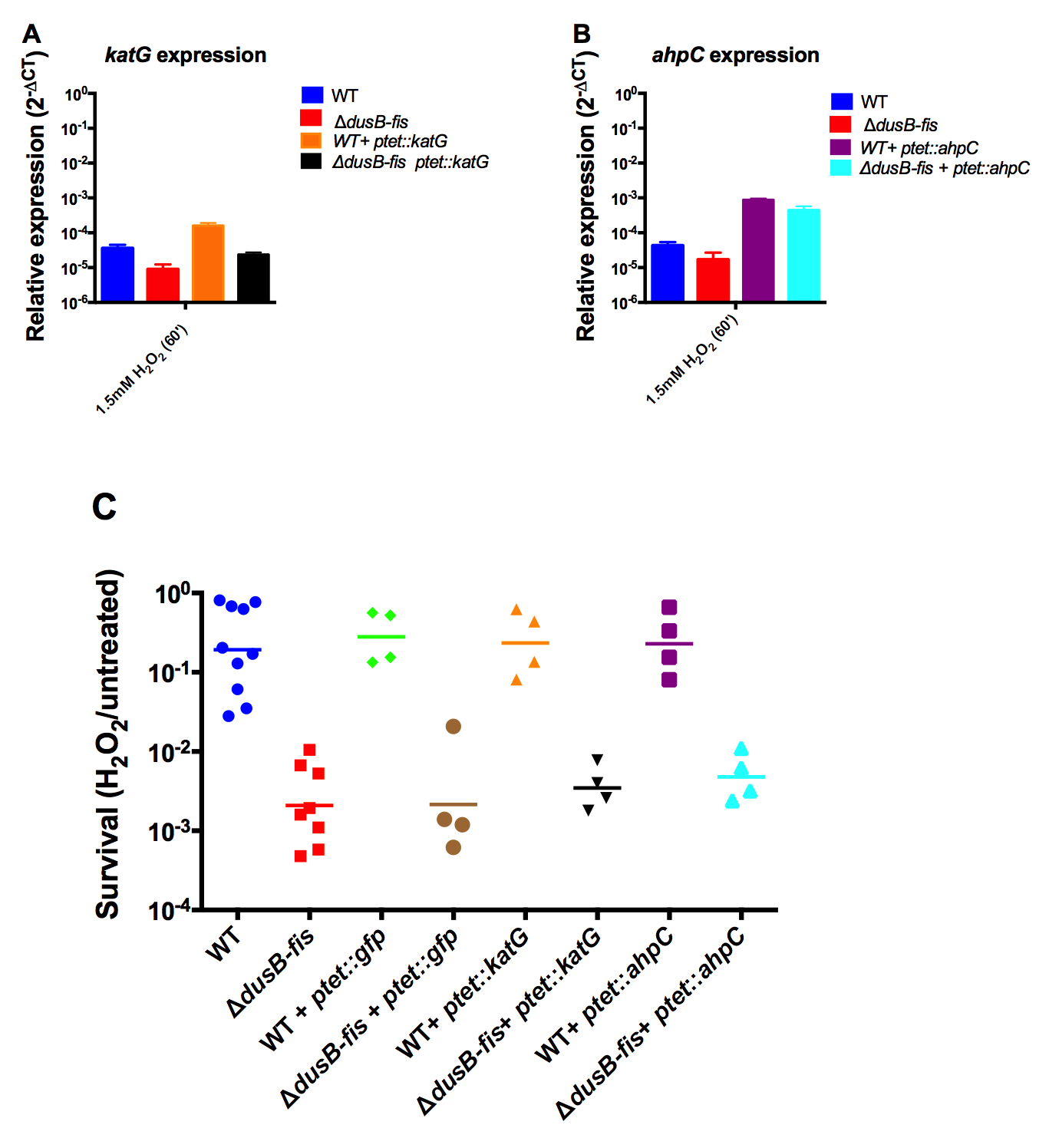

Supplement: S6 Fig — Exponential phase cultures were washed and diluted 1:50 into M9 glucose medium or M9 glucose containing 1.5mM H2O2 and incubated with aeration for 60 minutes. (A-B) Following treatment, RNA was isolated from samples exposed to H2O2, which was used to generate cDNA, and qPCR reactions were performed. Relative expression was determined by normalizing to 16S RNA using the ΔCT method. Bars represent the mean and standard error of 3–7 biological replicates. (C) A fraction of each H2O2-treated and untreated culture was also plated to determine surviving bacteria. Survival was calculated by determining the number of CFUs recovered following treatment divided by the number of CFUs recovered from untreated cultures. (TIFF) [file ppat.1005898.s006.tiff]

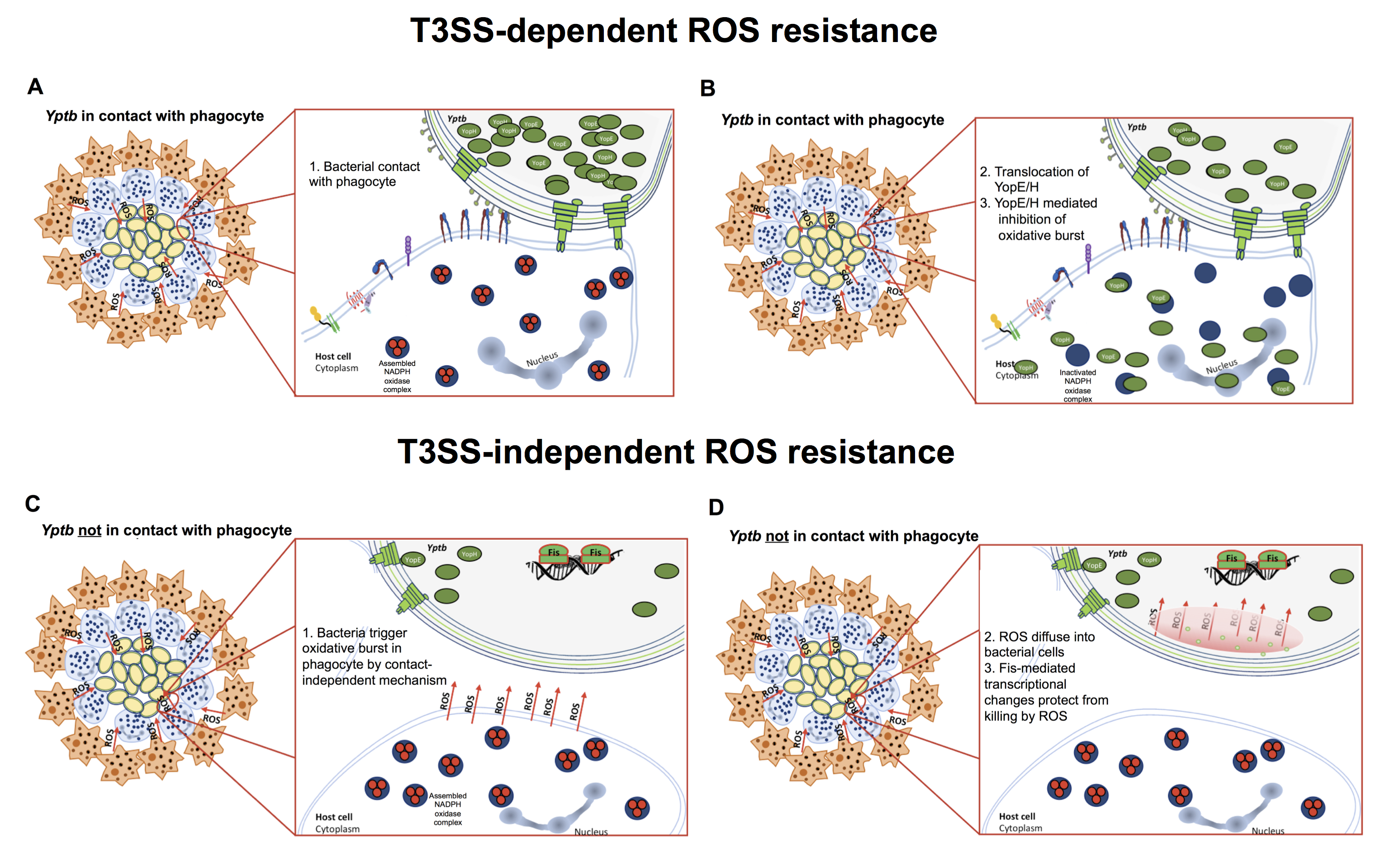

Supplement: S7 Fig — During growth within livers and spleens, Yptb forms extracellular aggregates or microcolonies [15,16]. Following stimulation of the immune response by infecting bacteria, neutrophils, macrophages, and inflammatory monocytes are recruited to sites of infection. (A) Some cells in close contact with bacteria on the periphery of the microcolony become translocated with T3SS effectors, (B) resulting in inhibition of NADPH oxidase activation and oxidative burst. (C) Other cells, which are not translocated with Yops, undergo oxidative burst in response to bacterial contact and/or PRR activation. (D) In WT infections, ROS released by these cells diffuse into the Yptb microcolony, where their bactericidal effects are resisted in a Fis-dependent manner, potentially through transcriptional induction of ROS-responsive genes. However, in Δfis infections, mutants are unable to resist the bactericidal effects of ROS and are killed or restricted for growth. (TIFF) [file ppat.1005898.s007.tiff]
