# Supplementary material for: Fis Is Essential for Yersinia pseudotuberculosis Virulence and Protects against Reactive Oxygen Species Produced by Phagocytic Cells during Infection
Source: PLoS Pathog. 2016 Sep 30;12(9):e1005898. doi: 10.1371/journal.ppat.1005898 (PMC5045184; doi:10.1371/journal.ppat.1005898)
Supplement: S1 Table — List of strains and plasmids that were constructed and/or utilized in this study. (DOCX) [file ppat.1005898.s008.docx]

| **Strains** | **Description** | **Source or Reference** |
| --- | --- | --- |
| ***E.coli*** |  |  |
| ERG5 | DH5α pRK600 | [1] |
| ERG73 | DH5αλpir pCVD442-Δ*aroA* | This study |
| ERG60 | DH5αλpir pCVD442-Δ*aroE* | This study |
| ERG63 | DH5αλpir pCVD442-Δ*purM* | This study |
| ERG99 | SY327λpir pCVD442-Δ*YPK_3179* | This study |
| ERG86 | DH5αλpir pCVD442-Δ*YPK_3184* | This study |
| ERG181 | SY327λpir pCVD442-Δ*YPK_3185* | This study |
| ERG78 | DH5αλpir pCVD442-Δ*rfaH* | This study |
| ERG79 | DH5αλpir pCVD442-Δ*wecC* | This study |
| ERG69 | DH5αλpir pCVD442-Δ*arnDT* | This study |
| ERG49 | DH5αλpir pCVD442-Δ*dusB-fis* | This study |
| ERG71 | DH5αλpir pCVD442-Δ*YPK_1920* | This study |
| ERG72 | SY327λpir pCVD442-Δ*oppD* | This study |
| ERG75 | DH5αλpir pCVD442-Δ*flgD* | This study |
| ERG76 | DH5αλpir pCVD442-Δ*YPK_2594* | This study |
| ERG41 | SY327λpir pCVD442-Δ*psaABCEF* | This study |
| ERG100 | SY327λpir pCVD442-Δ*YPK_3600* | This study |
| ERG87 | SY327λpir pCVD442-Δ*YPK_3656* | This study |
| ERG77 | DH5αλpir pCVD442-Δ*YPK_3765* | This study |
| ERG64 | DH5αλpir pCVD442-Δ*YPK_1604* | This study |
| ERG40 | SY327λpir pCVD442-Δ*YPK_2061* | This study |
| ERG261 | DH5αλpir pCVD442-*dusB-fis* | This study |
| ERG336 | DH5αλpir pCVD442-Δ*fis* | This study |
| CC99 | DH5αλpir pSR47S-ETEM | [2] |
| CC151 | DH5αλpir pSR47S-HTEM | [3] |
| ERG344 | DH5αλpir pACYC184-*ptet::katG* | This study |
| ERG389 | DH5αλpir pACYC184-*ptet:ahpC* | This study |
| ***Yersinia pseudotuberculosis*** | |  |
| ERG97 | IP2666 | [4] |
| ERG224 | IP2666 *yopH*-NdeI Kan^R^ | [5] |
| ERG318 | IP2666 *yopE::mcherry* | [6] |
| ERG169 | YPIII pIB1^-^  Δ*aroA* | This study |
| ERG115 | YPIII pIB1^-^  Δ*aroE* | This study |
| ERG113 | YPIII pIB1^-^  Δ*purM* | This study |
| ERG194 | YPIII pIB1^-^  Δ*YPK_3179* | This study |
| ERG96 | YPIII pIB1^-^  Δ*YPK_3184* | This study |
| ERG199 | YPIII pIB1^-^  Δ*YPK_3185* | This study |
| ERG110 | YPIII pIB1^-^  Δ*rfaH* | This study |
| ERG140 | YPIII pIB1^-^  Δ*wecC* | This study |
| ERG95 | YPIII pIB1^-^  Δ*arnDT* | This study |
| ERG121 | YPIII pIB1^-^  Δ*dusB-fis* | This study |
| ERG149 | YPIII pIB1^-^  Δ*YPK_1920* | This study |
| ERG174 | YPIII pIB1^-^  Δ*oppD* | This study |
| ERG114 | YPIII pIB1^-^  Δ*flgD* | This study |
| ERG193 | YPIII pIB1^-^  Δ*YPK_2594* | This study |
| ERG51 | YPIII pIB1^-^  Δ*psaABCEF* | This study |
| ERG205 | YPIII pIB1^-^  Δ*YPK_3600* | This study |
| ERG221 | YPIII pIB1^-^  Δ*YPK_3656* | This study |
| ERG147 | YPIII pIB1^-^  *ΔYPK_3765* | This study |
| ERG116 | YPIII pIB1^-^  Δ*YPK_1604* | This study |
| ERG56 | YPIII pIB1^-^  Δ*YPK_2061* | This study |
| ERG166 | IP2666 Δ*aroA* | This study |
| ERG124 | IP2666 Δ*aroE* | This study |
| ERG185 | IP2666 Δ*purM* | This study |
| ERG198 | IP2666 Δ*YPK_3179* | This study |
| ERG120 | IP2666 Δ*YPK_3184* | This study |
| ERG201 | IP2666 Δ*YPK_3185* | This study |
| ERG127 | IP2666 Δ*rfaH* | This study |
| ERG158 | IP2666 Δ*wecC* | This study |
| ERG186 | IP2666 Δ*arnDT* | This study |
| ERG146 | IP2666 Δ*dusB-fis* | This study |
| ERG187 | IP2666 Δ*YPK_1920* | This study |
| ERG164 | IP2666 Δ*oppD* | This study |
| ERG125 | IP2666 Δ*flgD* | This study |
| ERG162 | IP2666 Δ*YPK_2594* | This study |
| ERG148 | IP2666 Δ*psaABCEF* | This study |
| ERG207 | IP2666 Δ*YPK_3600* | This study |
| ERG211 | IP2666 Δ*YPK_3656* | This study |
| ERG163 | IP2666 *ΔYPK_3765* | This study |
| ERG179 | IP2666 Δ*YPK_1604* | This study |
| ERG222 | IP2666 Δ*YPK_2061* | This study |
| ERG316 | IP2666 *yopH*-NdeI Kan^R^ Δ*dusB-fis* | This study |
| ERG360 | IP2666 Δ*fis* | This study |
| ERG364 | IP2666 *yopH*-NdeI Kan^R^ Δ*fis* | This study |
| ERG301 | IP2666 Δ*dusB-fis::dusB-fis* | This study |
| ERG291 | IP2666 ETEM | [2] |
| ERG292 | IP2666 Δ*yscF-*ETEM | This study |
| FM034 | IP2666 Δ*yopB-*ETEM | [7] |
| ERG293 | IP2666 Δ*dusB-fis-*ETEM | This study |
| ERG332 | IP2666 HTEM | This study |
| ERG334 | IP2666 Δ*yscF-*HTEM | This study |
| ERG333 | IP2666 *yopH-*NdeI Δ*yopB-*HTEM | This study |
| ERG377 | IP2666 Δ*dusB-fis-*HTEM | This study |
| ERG400 | IP2666 Δ*yscF* | [8] |
| ERG376 | IP2666 *yopH*-NdeI Kan^R^ Δ*dusB*-*fis ΔyscF* | This study |
| ERG259 | IP2666 *Δhmp* | [9] |
| ERG247 | IP2666 pACYC184-*ptet::gfp* | [6] |
| ERG277 | IP2666 Δ*dusB-fis* pACYC184-*ptet::gfp* | This study |
| ERG370 | IP2666 pACYC184-*ptet::katG* | This study |
| ERG374 | IP2666 Δ*dusB-fis* pACYC184-*ptet::katG* | This study |
| ERG397 | IP2666 pACYC184-*ptet::ahpC* | This study |
| ERG398 | IP2666 Δ*dusB-fis* pACYC184-*ptet::ahpC* | This study |

**REFERENCES:**

1. Kessler B, de Lorenzo V, Timmis KN (1992) A general system to integrate lacZ fusions into the chromosomes of gram-negative eubacteria: regulation of the Pm promoter of the TOL plasmid studied with all controlling elements in monocopy. Mol Gen Genet 233: 293-301.

2. Harmon DE, Davis AJ, Castillo C, Mecsas J (2010) Identification and characterization of small-molecule inhibitors of Yop translocation in Yersinia pseudotuberculosis. Antimicrob Agents Chemother 54: 3241-3254.

3. Durand EA, Maldonado-Arocho FJ, Castillo C, Walsh RL, Mecsas J (2010) The presence of professional phagocytes dictates the number of host cells targeted for Yop translocation during infection. Cell Microbiol 12: 1064-1082.

4. Simonet M, Falkow S (1992) Invasin expression in Yersinia pseudotuberculosis. Infect Immun 60: 4414-4417.

5. Fisher ML, Castillo C, Mecsas J (2007) Intranasal inoculation of mice with Yersinia pseudotuberculosis causes a lethal lung infection that is dependent on Yersinia outer proteins and PhoP. Infect Immun 75: 429-442.

6. Crimmins GT, Mohammadi S, Green ER, Bergman MA, Isberg RR, et al. (2012) Identification of MrtAB, an ABC transporter specifically required for Yersinia pseudotuberculosis to colonize the mesenteric lymph nodes. PLoS Pathog 8: e1002828.

7. Maldonado-Arocho FJ, Green C, Fisher ML, Paczosa MK, Mecsas J (2013) Adhesins and host serum factors drive Yop translocation by yersinia into professional phagocytes during animal infection. PLoS Pathog 9: e1003415.

8. Davis AJ, Mecsas J (2007) Mutations in the Yersinia pseudotuberculosis type III secretion system needle protein, YscF, that specifically abrogate effector translocation into host cells. J Bacteriol 189: 83-97.

9. Davis KM, Mohammadi S, Isberg RR (2015) Community behavior and spatial regulation within a bacterial microcolony in deep tissue sites serves to protect against host attack. Cell Host Microbe 17: 21-31.
