# Supplementary material for: Fis Is Essential for Yersinia pseudotuberculosis Virulence and Protects against Reactive Oxygen Species Produced by Phagocytic Cells during Infection
Source: PLoS Pathog. 2016 Sep 30;12(9):e1005898. doi: 10.1371/journal.ppat.1005898 (PMC5045184; doi:10.1371/journal.ppat.1005898)
Supplement: S2 Table — List of primers utilized in this study for generation of pCVD442 and pACYC184 plasmids, preparation and sequencing of Illumina libraries, and qRT-PCR analysis. (DOCX) [file ppat.1005898.s009.docx]

| **Primer name** | **Primer sequence** |
| --- | --- |
| *aroA* p1 | 5'-ATTATCTAGACATTGACGGCGTTGCGATTAATGAACAAC-3' |
| *aroA* p2 | 5'ACAGCGTCGACCTTGAGAATAGGAACTTCGGAATAGGAACTCATGGGAAAAACATTCTCCAGTCTTGTATAG-3' |
| *aroA* p3 | 5'-TTCCTATTCTCAAGGTCGACGCTGTATTTAAGTGGTCCAACTGATAGCACCTGAATTGAATGGGTTCTATCACA-3' |
| *aroA* p4 | 5'-ATTA GAGCTCTGCAACTGTAGCATACGTCTGTGTGC-3' |
| *aroE* p1 | 5'-ATTATCTAGACAAGGGAAACTGTTACAACGTAAGTCTC-3' |
| *aroE* p2 | 5'-ACAGCGTCGACCTTGAGAATAGGAACTTCGGAATAGGAACTCATACAATATCCTACCCTTGGCGAAAC-3' |
| *aroE* p3 | 5'-TTCCTATTCTCAAGGTCGACGCTGTATTTAAGTGGTCCAACGGTAAATAGAAGCACACACCAATGAATGC-3' |
| *aroE* p4 | 5'-ATTAGCATGCGATGCGCATTATAGGGAGTCGATTTTTTTG-3' |
| *purM* p1 | 5'-ATTATCTAGATGGTCATATGGGCCGAACCGGA-3' |
| *purM* p2 | 5'-ACAGCGTCGACCTTGAGAATAGGAACTTCGGAATAGGAACTCATAATCTAAGTTTCTCTCGTCTTCGAAGC-3' |
| *purM* p3 | 5'-TTCCTATTCTCAAGGTCGACGCTGTATTTAAGTGGTCCAACTTAGGTTAATAAGTTAAAGTGAGAAGGGTATTGAAG-3' |
| *purM* p4 | 5'-ATTAGCATGCCTGCAGAACATGGAATGATCACATTGAATC-3' |
| *YPK_3179* p1 | 5'-ATTATCTAGAATTGCAAAAGTATTTTTCGATAATACTGTGGATGCTTG-3' |
| *YPK_3179* p2 | 5'-ACAGCGTCGACCTTGAGAATAGGAACTTCGGAATAGGAACTCATATATATTCTCTTTATGACTTTAGAGTAAATCAGTAGTGAC-3' |
| *YPK_3179* p3 | 5'-TTCCTATTCTCAAGGTCGACGCTGTATTTAAGTGGTCCAACTAATGTTAATATAATAACTTGGGAGGATAATATTTAATTTTAAAGTTGG-3' |
| *YPK_3179* p4 | 5'-ATTAGCATGCGGATTAATATAGTTAATTAAGTGTTCAACATAGTCATTAATAATAGATATTTTT-3' |
| *YPK_3184* p1 | 5'-ATTATCTAGAGTAGTTCTATATTCAATATTGCAAATATCCAAAGGC-3' |
| *YPK_3184* p2 | 5'-ACAGCGTCGACCTTGAGAATAGGAACTTCGGAATAGGAACTCATTTCTCACCAACCAACTATTTTATTTAGCATTAGAA-3' |
| *YPK_3184* p3 | 5'-TTCCTATTCTCAAGGTCGACGCTGTATTTAAGTGGTCCAACAACAAATAAAATGGATTACAATATATGACTAAGATTGCGCT-3' |
| *YPK_3184* p4 | 5'-ATTAGCATGCAACGGTAATTTGCTTGCCTGTTGCAATAAC-3' |
| *YPK_3185* p1 | 5'-ATTATCTAGATTAGGGCCAGCGGCTATAATTCCATTTG-3' |
| *YPK_3185* p2 | 5'ACAGCGTCGACCTTGAGAATAGGAACTTCGGAATAGGAACTCAAAGTTAACTCCAATTAAAATAAAACATCACATTATTCTTATACCG-3' |
| *YPK_3185* p3 | 5'TTCCTATTCTCAAGGTCGACGCTGTATTTAAGTGGTCCAACTAAAATAGTTGGTTGGTGAGAAATGAAGATAATTTACGA-3' |
| *YPK_3185* p4 | 5'ATTAGCATGCTCGCTTCTAATATTGGAATACCAAAACCTTCATAG-3' |
| *rfaH* p1 | 5'-ATTATCTAGACCAATACCTGCTGGCGATAGATGC-3' |
| *rfaH* p2 | 5'-ACAGCGTCGACCTTGAGAATAGGAACTTCGGAATAGGAACTCATAGTTTCACATACCTTTGGGTACTATTTAGCA-3' |
| *rfaH* p3 | 5'-TTCCTATTCTCAAGGTCGACGCTGTATTTAAGTGGTCCAACTAATAACACGCTGACTTACAATAATCTGGATGA-3' |
| *rfaH* p4 | 5'-ATTACCCGGGTGCGTTGAAAGATGCAGTTCCTGAGCTA-3' |
| *wecC* p1 | 5'-ATTATCTAGACATACCTGTCGGCCATGTCGAG-3' |
| *wecC* p2 | 5'-ACAGCGTCGACCTTGAGAATAGGAACTTCGGAATAGGAACTACTCATAGCGTCACCTGATGATTCTTTAAAG-3' |
| *wecC* p3 | 5'-TTCCTATTCTCAAGGTCGACGCTGTATTTAAGTGGTCCAACTGGCGTTGAGGCGAATTCTCGTCA-3' |
| *wecC* p4 | 5'-ATTAGCATGCCGCAAATGGTTTCAACTACTTCAATATTCTTGC-3' |
| *arnDT* p1 | 5'-ATTAATCTAGACTATTGCGACACCCATTGAATACACCC-3' |
| *arnDT* p2 | 5'-ACAGCGTCGACCTTGAGAATAGGAACTTCGGAATAGGAACTCATGCGTTCAACTCATCTTTAGGGGCT-3' |
| *arnDT* p3 | 5'-TTCCTATTCTCAAGGTCGACGCTGTATTTAAGTGGTCCAACTGAACAGCTACCTACTCTTGCCGATG-3' |
| *arnDT* p4 | 5'-ATTAGCATGCGCTGGCGTGGTACATCGTTACGG-3' |
| *dusB-fis* p1 | 5'-ATTAGCATGCCCAATATTCTGGCAGGCCCATTACG-3' |
| *dusB-fis* p2 | 5’-ACAGCGTCGACCTTGAGAATAGGAACTTCGGAATAGGAACTCATAGACAAAGAATGACCACACTGTGTCC-3’ |
| *dusB-fis* p3 | 5’-TTCCTATTCTCAAGGTCGACGCTGTATTTAAGTGGTCCAACTGATACTAGTCAGTTAACTTGTTGTTTAAAAAGGCAC-3’ |
| *dusB-fis* p4 | 5'- ATTAGAGCTCAAAGCTACGATGATATCGTTGGCTTGTTGAT-3' |
| *YPK_1920* p1 | 5'-ATTATCTAGATGTCATGTCGTATCGTTTACAGAACCATC-3' |
| *YPK_1920* p2 | 5'-ACAGCGTCGACCTTGAGAATAGGAACTTCGGAATAGGAACTCATTCCTTCGCCCTCGTCGGTATT-3' |
| *YPK_1920* p3 | 5'-TTCCTATTCTCAAGGTCGACGCTGTATTTAAGTGGTCCAACCTTTTCTAAGGAACCATCATGAAAAAGCATACTTTA-3' |
| *YPK_1920* p4 | 5'-ATTAGAGCTCTCTTCGTATTTATGGAAAGTTGGCACCAAG-3' |
| *oppD* p1 | 5'-ATTATCTAGAGGGCTGTTGGCCAGTAATGGTCG-3' |
| *oppD* p2 | 5'-ACAGCGTCGACCTTGAGAATAGGAACTTCGGAATAGGAACTCATACTAATTCCCCTACCGGCTTATAACAG-3' |
| *oppD* p3 | 5'-TTCCTATTCTCAAGGTCGACCTGTATTTAAGTGGTCCAACTAACTTGGTGATTTTCTGTGATTACGTCTGCAT-3' |
| *oppD* p4 | 5'-ATTAGCATGCCTGGGAACATATTTTTAGTTTCCGCAACCATC-3' |
| *flgD* p1 | 5'-ATTATCTAGAAGCGCTTAATTTGCGTGCACAACGG-3' |
| *flgD* p2 | 5'-ACAGCGTCGACCTTGAGAATAGGAACTTCGGAATAGGAACTCATTATGCTGGCTCCTGTTATTGACCC-3' |
| *flgD* p3 | 5'-TTCCTATTCTCAAGGTCGACGCTGTATTTAAGTGGTCCAACTTATAAGTCTGTTGTTAGGCTTTCATAGAACTGTTA-3' |
| *flgD* p4 | 5'-ATTAGCATGCTTGATTTCATGGCGGTTACCCAAGCTAT-3' |
| *YPK_2594* p1 | 5'-ATTATCTAGACACTGCTGTCAGTATATGCAACATAAACC-3' |
| *YPK_2594* p2 | 5'-ACAGCGTCGACCTTGAGAATAGGAACTTCGGAATAGGAACTCAAGCTGTCCTCATGCCACACCA-3' |
| *YPK_2594* p3 | 5'-TTCCTATTCTCAAGGTCGACGCTGTATTTAAGTGGTCCAACGAGAAATAAGGGAATATCATTGTGAATAAGAAAAACAG-3' |
| *YPK_2594* p4 | 5'-ATTAGCATGCTATCTTCAGGCC AGTGAATAGTATTGATCG-3' |
| *psaABCEF* p1 | 5'-GGTTAAAAAGGATCGATCCTCTAGAATGGGCGGCCCAGTGAATAAAGCG-3' |
| *psaABCEF* p2 | 5'-ACAGCGTCGACCTTGAGAATAGGAACTTCGGAATAGGAACTCATTTGCCCTCACCTCCCCTGAT-3' |
| *psaABCEF* p3 | 5'-TTCCTATTCTCAAGGTCGACGCTGTATTTAAGTGGTCCAACTAAAGGCATCCGCGATGACACAATTCC-3' |
| *psaABCEF* p4 | 5'-CCGGGAGAGCTCGATATCGCATGCTGAGAAATTTAAACTCGCGTGTGAGAAGTACG-3' |
| *YPK_3600* p1 | 5'-ATTATCTAGAATCAGTTTCAGAATTTCCAAACCAATATTCACTGC-3' |
| *YPK_3600* p2 | 5'-ACAGCGTCGACCTTGAGAATAGGAACTTCGGAATAGGAACTCATCATGACATCCTATTTATACCGTCATACTTC-3' |
| *YPK_3600* p3 | 5'-TTCCTATTCTCAAGGTCGACGCTGTATTTAAGTGGTCCAACCAGCACTAAAATATGTCTGTAAGTGACACCA-3' |
| *YPK_3600* p4 | 5'-ATTAGCATGCTATTTTTAGCCTCGTGAGTGAAGGGTTGA-3' |
| *YPK_3656* p1 | 5'-ATTATCTAGAGGGGCGCAATATTTTTCAGTCAGATGCGC-3' |
| *YPK_3656* p2 | 5'-ACAGCGTCGACCTTGAGAATAGGAACTTCGGAATAGGAACTCATAGAAACTCCTGTTTTATATGGTGGTCACA-3' |
| *YPK_3656* p3 | 5'-TTCCTATTCTCAAGGTCGACGCTGTATTTAAGTGGTCCAACTTGAAGTAATCCGATAAGGGTCACTGG-3' |
| *YPK_3656* p4 | 5'-ATTACCCGGGGTCCCCGGCACTCCTACGC-3' |
| *YPK_3765* p1 | 5'-ATTATCTAGACTTCGTTGATGGAATACATGCAACCGG-3' |
| *YPK_3765* p2 | 5'-ACAGCGTCGACCTTGAGAATAGGAACTTCGGAATAGGAACTCATAAATCTTCCGTATTAATAAGCTTAAAATTTGTAGCC-3' |
| *YPK_3765* p3 | 5'-TTCCTATTCTCAAGGTCGACGCTGTATTTAAGTGGTCCAACTTGGATTAGGCTGGATAAAATATCCCCTG-3' |
| *YPK_3765* p4 | 5'-ATTAGAGCTCTTCGCACGTTTAGTCAAAATCACTTATTCAATTG-3' |
| *YPK_1604* p1 | 5'-ATTATCTAGAACTGGTCGGTGCACAAGGTATTACCT-3' |
| *YPK_1604* p2 | 5'ACAGCGTCGACCTTGAGAATAGGAACTTCGGAATAGGAACTCATTGATGGCTCCTCCTATCAGGTGA- 3' |
| *YPK_1604* p3 | 5'TTCCTATTCTCAAGGTCGACGCTGTATTTAAGTGGTCCAACTAACCCGAGCCATCGGAGGCCAT-3' |
| *YPK_1604* p4 | 5'-ATTAGCATGCGCAATATAAGGGGTTATCTTTGAGCATGGG-3' |
| *YPK_2061*p1 | 5'-ATTATCAGATTTACTCCTCTGCCTATCCTCACCGTT-3' |
| *YPK_2061* p2 | 5'-ACAGCGTCGACCTTGAGAATAGGAACTTCGGAATAGGAACTCATAATGATTCCATTTATTAAAAGTTAATTTTTGATAAGGCAGGA-3' |
| *YPK_2061* p3 | 5'-TTCCTATTCTCAAGGTCGACGCTGTATTTAAGTGGTCCAACTAATATTCTGTTGATTAACGCAGATAACCAGAATGAC-3' |
| *YPK_2061* p4 | 5'-ATTAGCATGCACGCAAAAGTATTGAGATAGACTATTGTCCAAATTG-3' |
| *fis* p1 | 5'-GATGGGTTAAAAAGGATCGATCCTCTAGACCGGGATTCGTAACGTGC-3' |
| *fis* p2 | 5'-AGTTCTGTCAGCTCTTTTTTCT-3' |
| *fis* p3 | 5'-AGAAAAAAGAGCTGACAGAACTTACTAGTCAGTTAACTTGTTGTT-3' |
| *fis* p4 | 5'-CGGGAGAGCTCGATATCGCATGCATGATATCGTTGGCTTGTTGATATC-3' |
| mini-TnSeq F1 | 5'-AGTTCCTATTCCGAAGTTCCTATTCTCAAG-3' |
| mini-TnSeq R1 | 5'-GTGACTGGAGTTCAGACGTGTGCTCTTCCGATCTGGGGGGGGGGGGGGGG-3' |
| mini-TnSeq F2 | 5'-CAAGGTCGACGCTGTATTTAAGTGGTCCAACT-3' |
| mini-TnSeq R2 | 5'-CAAGCAGAAGACGGCATACGAGATNNNNNNGTGACTGGAGTTCAGACGTGTGCTCTTCCGATCT-3' |
| mini-TnSeq Ilumina sequencing primer | 5'-CAAGGTCGACGCTGTATTTAAGTGGTCCAACT-3' |
| *ptet::katG* F | 5'-TGCGGGATCTCTAGATTTAAGAAGGAGATATACATATGTTAAAAAAAATCTTACCCGTAC-3' |
| *ptet*::*katG* R | 5'-GCATGCCTGCAGGTCTGGACATTTATTAGTTATTTTTTATATCAAAGCGA-3' |
| *ptet::ahpC* F | 5'-TGCGGGATCTCTAGATTTAAGAAGGAGATATACATATGGTTCTGGTAACTCGTCAA-3' |
| *ptet*::*ahpC* R | GGAATGGTCTGCAGGTCTGGACATTTATTACAGCTTAGATGCGTTCTC-3' |
| 16S RNA qPCR F | 5'-CAGCCACACTGGAACTGAGA-3' |
| 16S RNA qPCR R | 5'-GTTAGCCGGTGCTTCTTCTG-3' |
| *ahpC* qPCR F | 5'-CGTGGTGTTGAAGTTGTTGG-3' |
| *ahpC* qPCR R | 5'-TGTCGATCAGGAATGAACCA-3' |
| *katG* qPCR F | 5'-GAAATGCAGCCATTGAGGAT-3' |
| *katG*  qPCR R | 5'-TAGGCGAAAGCGTGTTTCTT-3' |
| *grxA* qPCR F | 5'-TTACTGTGTCCGTGCCAAAGAG-3' |
| *grxA* qPCR R | 5'-CGTAGGCTTCGAAATCAGT-3' |
| *recA* qPCR F | 5'-CCCAGAATCATCAGGTAAGAC-3' |
| *recA* qPCR R | 5'-TCAGCGCATCACAAATTTCCA-3' |
| *rpoC* qPCR F | 5'-ACGGGTAGCGGTAAAGACCT-3' |
| *rpoC* qPCR R | 5'-AATACGCGCCAAGGTATCAC-3' |
